# Identification of the natural chalcone glycoside hydroxysafflor yellow A as a suppressor of P53 overactivation‐associated hematopoietic defects

**DOI:** 10.1002/mco2.352

**Published:** 2023-08-24

**Authors:** Jing Chen, Can Ren, Chong Yao, Mirko Baruscotti, Yi Wang, Lu Zhao

**Affiliations:** ^1^ Pharmaceutical Informatics Institute, College of Pharmaceutical Sciences Zhejiang University Hangzhou China; ^2^ Huzhou Central Hospital, Affiliated Huzhou Hospital Zhejiang University School of Medicine Huzhou China; ^3^ Department of Biosciences University of Milano Milan Italy; ^4^ Innovation Institute for Artificial Intelligence in Medicine of Zhejiang University Hangzhou China; ^5^ National Key Laboratory of Chinese Medicine Modernization, Innovation Center of Yangtze River Delta Zhejiang University Jiaxing China

**Keywords:** chalcone glycoside, hematopoiesis, hydroxysafflor yellow A, P53 signaling, zebrafish phenotypic screening, zinc deficiency

## Abstract

Enhanced P53 signaling may lead to hematopoietic disorders, yet an effective therapeutic strategy is still lacking. Our study, along with previous research, suggests that P53 overactivation and hematopoietic defects are major consequences of zinc deficiency. However, the relationship between these two pathological processes remains unclear. In this study, we observed a severe reduction in the number of hematopoietic stem cells (HSCs) and multi‐lineage progenitor cells in zebrafish treated with the zinc chelator *N*,*N*,*N*′,*N*′‐tetrakis(2‐pyridylmethyl)ethylenediamine and showed the indispensable role of P53 signaling in the process. Next, we took advantage of HSCs‐labeled transgenic zebrafish and conducted a highly efficient phenotypic screening for small molecules against P53‐dependent hematopoietic disorders. Hydroxysafflor yellow A (HSYA), a natural chalcone glycoside, exhibited potent protection against hematopoietic failure in zinc‐deficient zebrafish and strongly inhibited the P53 pathway. We confirmed the protective effect of HSYA in zinc‐deficient mice bone marrow nucleated cells, which showed a significant suppression of P53 signaling and oxidative stress. Furthermore, the hematopoietic‐protective activity of HSYA was validated using a mice model of myelotoxicity induced by 5‐FU. In summary, our work provides an effective phenotypic screening strategy for identifying hematopoietic‐protective agents and reveals the novel role of HSYA as a promising lead compound in rescuing hematopoietic disorders associated with P53 overactivation.

## Graphical abstract

Our work elucidates the critical role of p53 activation in zinc‐deficiency associated hematopoietic disorders and suggests an effective phenotypic screening strategy for hematopoietic‐protective agents; we also discovered the novel role of the chalcone glycoside HSYA as a promising lead compound in rescuing P53 overactivation related hematopoietic disorders.

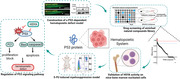

## 1 INTRODUCTION

Hematopoiesis refers to the continuous differentiation and formation of all blood cell lineages throughout the life cycle. The homeostasis of hematopoiesis needs precise regulation by various signaling pathways. Disruption in these pathways, whether caused by gene mutations or nongenetic risk factors such as irradiation, chemical exposures, nutritional deficiencies, and aging, can lead to a wide range of hematological disorders. In the hematopoietic system, the P53 protein plays a crucial role in maintaining the self‐renewal and quiescence of hematopoietic stem cells (HSCs).^1^ Studies in *P53*‐null mice have shown an expansion of the HSCs pool; however, their repopulation capabilities are compromised, as evidenced by competitive bone marrow transplantation.^2^, ^3^ On the other hand, excessive P53 activity beyond basal level can be detrimental to hematopoiesis. Mice with constitutive P53 expression or ionizing radiation‐induced P53 overexpression have exhibited drastic depletion of HSC regenerative capacity and hematopoietic failure. This is attributed to increased apoptosis and cell cycle arrest in HSCs and blood progenitor cells.^4^, ^5^ Additionally, studies have indicated that HSCs fail to increase with age in old mice with hyperactivated P53 expression compared to those with normal P53 levels.^6^ Hence, the level of P53 activity plays a critical role in determining hematopoietic homeostasis and requires precise control.

The expression of P53 signaling is typically maintained at low levels under normal conditions but can be upregulated by various factors. Recent studies have suggested a close association between P53 overactivation and the aging process.^7^, ^8^, ^9^ For instance, increased levels of p53 protein have been observed in astrocytes, fibroblasts, and retinal pigment epithelial cells isolated from elderly individuals.^10^ Environmental factors, such as oxidative stress or nutritional deficiency, may also contribute to enhanced P53 activity. In our previous study using a macrophage‐specific zinc importer protein *Slc39a10* knockout mouse model, we found that zinc deficiency led to enhanced P53 protein stability and increased macrophage apoptosis in response to inflammatory stimuli.^11^ Additionally, overexpressed P53 may also occur as a result of pharmacological reactivation in some cancer therapies.^12^ In all of these situations, hematopoiesis dysfunction or bone marrow suppressions have been observed.^13^, ^14^, ^15^ However, targeting P53 overactivation‐related hematopoietic defects specifically, without causing other detrimental problems such as an increased risk of tumorigenesis or affecting the antitumor efficacy of cancer therapy, remains a challenge.

Zebrafish has emerged as a rapidly growing animal model in new drug discovery and is considered the most suitable vertebrate model system for high‐throughput drug screening.^16^ The hematopoietic processes and related molecular mechanisms are highly conserved between zebrafish and mammals.^17^ Research on zebrafish hematopoietic disease models has significantly contributed to our understanding of human diseases and the discovery of new therapeutic strategies. Forward genetic screenings in zebrafish have identified multiple mutants with hematopoietic defects, such as cloche,^18^ riesling (beta‐spectrin gene mutation),^19^ and weh (ferroportin 1 mutation).^20^ Prohema, a derivative of prostaglandin E2 that promotes HSC mobilization, was initially identified through a zebrafish screen and is currently undergoing clinical trials for the treatment of leukemia or lymphoma patients undergoing umbilical cord blood transplantation.^21^ Moreover, the use of transgenic zebrafish lines with fluorescence‐labeled blood cells allows for the direct observation of the number and distribution of HSCs and downstream blood lineages in fish embryos using fluorescence microscopy. Combined with the advantages of small size and high fertility, the zebrafish model system greatly facilitates high‐throughput phenotypic drug screening at the animal level.

Hydroxysafflor yellow A (HSYA) is a quinochalcone C‐glycosides initially isolated from *Carthamus tinctorius* L. in 1993.^22^ Like many chalcone derivatives, HSYA is well known for its antioxidant properties, primarily due to the phenolic group in its molecular structure, which facilitates effective scavenging of reactive oxygen species (ROS).^23^ Additionally, HSYA has been reported to exhibit antioxidant capacity by mediating heme oxygenase‐1 expression and Nrf2 signaling.^24^ Its therapeutic effects mainly focus on cardiovascular and cerebrovascular diseases, such as ischemia/reperfusion myocardial injury, cardiac hypertrophy, hypertension, and cerebral ischemia. In addition to its antioxidant capacity, HSYA has potential pharmacological mechanisms involving anti‐inflammation, anti‐coagulation, and vasorelaxation.^25^

In this study, we designed a highly efficient in vivo drug screening strategy to discover hematopoietic‐protective agents. By utilizing both zebrafish and mouse model systems, we report for the first time the novel activity of HSYA in attenuating hematopoietic defects associated P53 overactivation. Further analysis revealed the potential roles of HSYA in regulating the P53 signaling pathway.

## 2 RESULTS

### 2.1 Zinc chelator TPEN induces P53‐dependent hematopoietic defects in zebrafish

**FIGURE 1:**
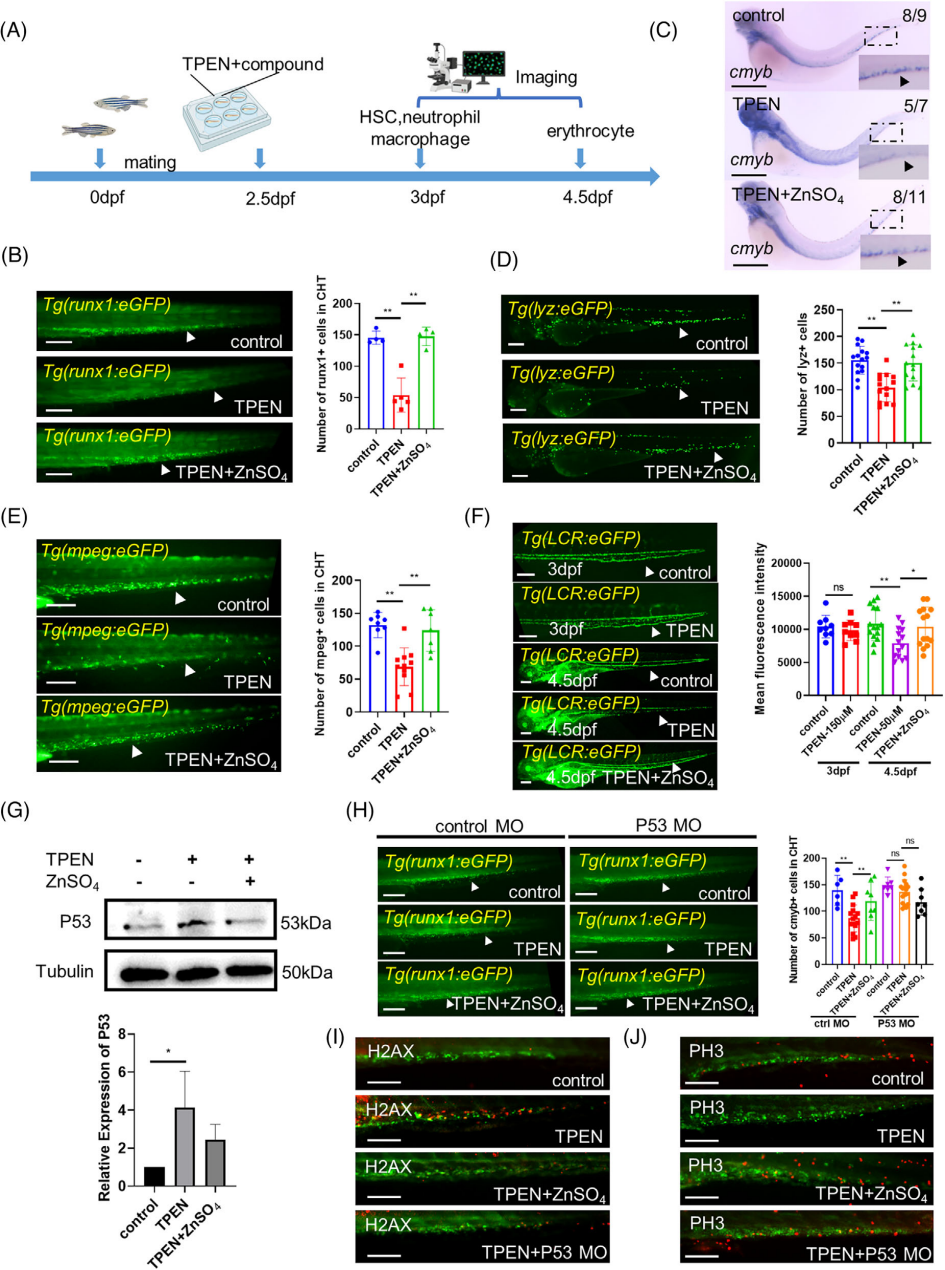
*N*,*N*,*N*′,*N*′‐tetrakis(2‐pyridylmethyl)ethylenediamine (TPEN)‐induced P53‐dependent hematopoietic defects in zebrafish: (A) timeline of TPEN modeling and observation time points for all hematopoietic lineages; (B) representative images of hematopoietic stem cells (HSCs) in control and TPEN (150 μM)‐treated *Tg(runx1: eGFP)* embryos with or without zinc incubation (100 μM) for 12 h. The quantitative results are shown in the right panel; (C) whole in situ hybridization showing the transcriptional expression of *cmyb*. The numbers at the top‐right corner show the number of embryos with representative phenotypes/the total number of embryos in the group. Positive stained cells are pointed by a black arrowhead in the zoomed inset. (D and E) Representative images of neutrophils in *Tg(lyz:eGFP)* embryos (D) and macrophages in *Tg(mpeg:eGFP)* embryos (E). The quantitative results are shown in the right panels; (F) representative images of red blood cells in *Tg(LCR:eGFP)* embryos at 3 and 4.5 dpf, respectively; (G) Western blot images and quantitative results showing P53 expression; (H) representative images and quantitative results showing the effect of *tp53* morpholino microinjection on TPEN‐induced HSCs reduction compared to that of standard control morpholino; (I and J) immunostaining of H2AX‐labeled apoptotic cells (red signals in I) and PH3‐labeled of proliferating cells (red signals in J) in HSCs labeled (green signals) *Tg(runx1: eGFP)* zebrafish with different treatments. At least 7 embryos were included in each group; for Western blot experiments, 30 embryos in each group from three separate experiments were used; arrowheads in (B–F, H) pointed to the positive stained cell signals; scale bar (in B, D–F, H), 200 μm; scale bar (in C), 500 μm; scale bar (in I–J), 100 μm. hpf, hours post fertilization. *p < 0.05; **p < 0.01.

Defective hematopoiesis of multiple blood lineages has been observed in mouse models fed with a zinc‐deficient diet.^15^, ^26^ To assess the impact of zinc deficiency on zebrafish hematopoiesis, we used *N*,*N*,*N*′,*N*′‐tetrakis(2‐pyridylmethyl)ethylenediamine (TPEN), a membrane‐permeable zinc chelator, to create a low‐zinc environment for zebrafish embryos (Figure 1A). In *Tg(runx1: eGFP)* zebrafish lines, which have green fluorescent‐labeled HSCs, the signal in the caudal hematopoietic tissue (CHT) was significantly reduced after 12 h of stimulation with 150 μM TPEN. Simultaneous zinc supplementation fully rescued the effect of TPEN (Figure 1B). Whole‐mount in situ hybridization also demonstrated a substantial reduction in the expression of *cmyb*, an essential transcriptional factor for HSCs maintenance, following TPEN treatment (Figure 1C).

We further investigated whether downstream hematopoietic lineages were also affected by zinc deficiency. After 12 h of TPEN treatment, both neutrophils and macrophages were greatly reduced, whereas erythrocytes were not significantly affected (Figure 1D–F). As a large proportion of red blood cells circulating in 3 day‐post‐fertilization (dpf) embryos are likely derived from the primitive hematopoietic wave, we extended TPEN treatment to 48 h at a lower concentration of 50 μM and observed a reduction in the number of erythrocytes at 4.5 dpf (Figure 1F). Zinc supplementation was able to restore all the aforementioned hematopoietic defective phenotypes.

Previous studies have indicated that P53 overexpression plays a significant role in zinc deficiency‐related diseases in various systems.^27^, ^28^ Hence, we aimed to investigate whether the TPEN‐induced hematopoietic defect was also dependent on P53. Following TPEN treatment, the expression level of P53 was found to be elevated compared to control embryos (Figure 1G). To further elucidate the involvement of P53 in zinc deficiency‐mediated hematopoietic disorders, we conducted *tp53* morpholino microinjections to knock down P53 expression in zebrafish embryos. Notably, no significant difference in the number of HSCs was observed between embryos injected with *tp53* morpholino and those injected with control morpholino. However, upon P53 expression blockage, TPEN was no longer able to induce hematopoietic defects. This observation strongly suggests that P53 function is essential in zinc deficiency‐mediated hematopoietic disorders (Figure 1H). Additionally, through whole‐embryo immunofluorescence staining, we detected increased expression of the DNA double‐strand breaks marker H2A.X and decreased expression of the mitosis marker PH3 in GFP‐labeled HSCs of TPEN‐treated zebrafish. Both of these changes were effectively rescued by zinc supplementation or *tp53* morpholino microinjection (Figure 1I,J). These findings indicate that zinc‐deficiency‐induced P53 overactivation disrupts the homeostasis of HSCs apoptosis and proliferation. Consequently, we established a zebrafish model of hematopoietic disorder with multi‐lineage defects using TPEN treatment and demonstrated the crucial role of P53 in this disease process.

### 2.2 Phenotypic screening in a P53‐dependent hematopoietic defective zebrafish model

**FIGURE 2:**
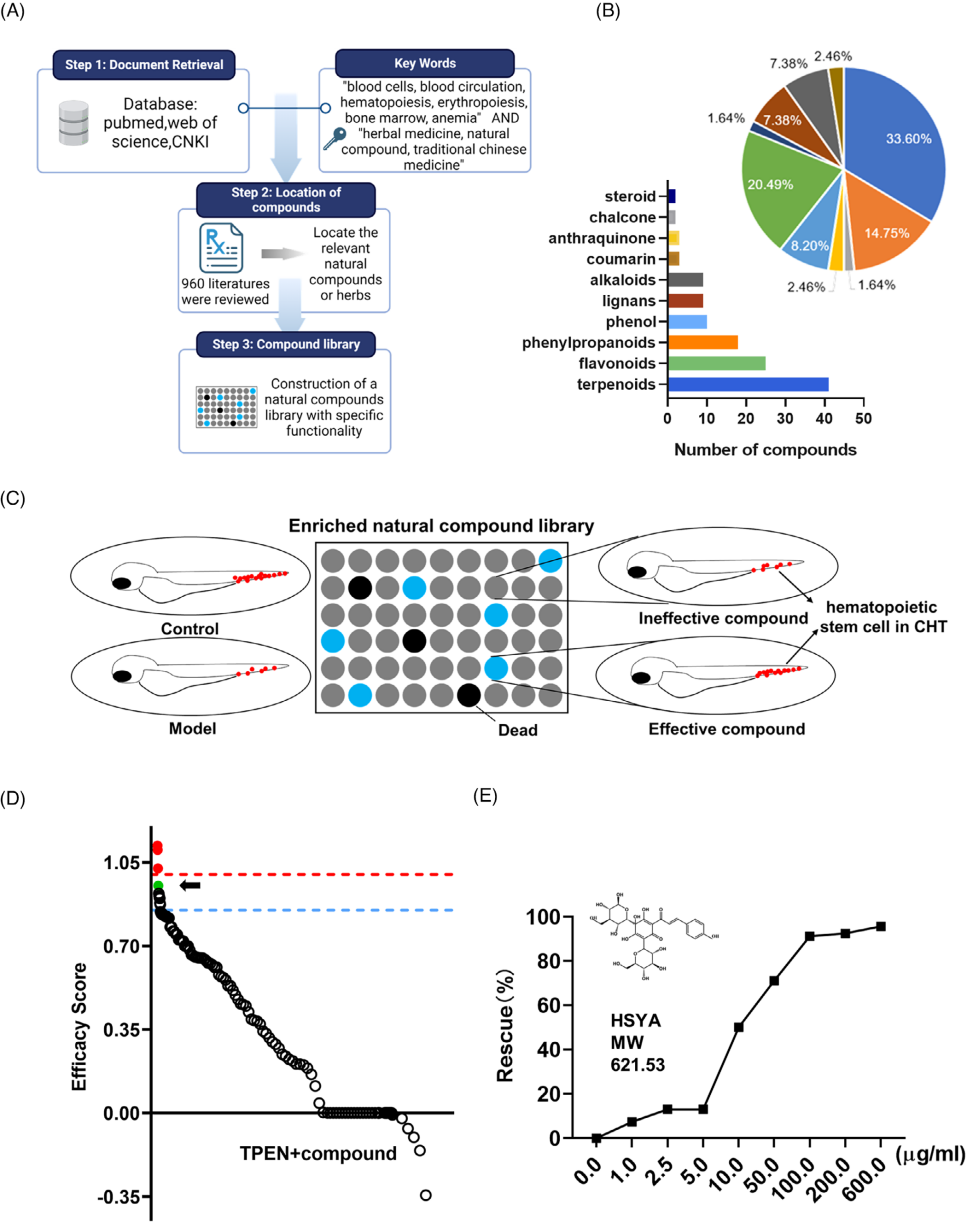
Phenotypic screening for hematopoietic‐protective compounds in *N*,*N*,*N*′,*N*′‐tetrakis(2‐pyridylmethyl)ethylenediamine (TPEN)‐treated zebrafish: (A) construction of an enriched library of natural compounds related to blood diseases; (B) chemical profile of the enriched natural compound library; (C) schematic diagram of phenotypic screening. The number of hematopoietic stem cells (HSCs) (represented by red dots) in the caudal hematopoietic tissue of zebrafish was used as a screening indicator; (D) the scatter plot summarizes the effect of all compounds on the number of HSCs. Efficacy score was calculated by (*N*_drug_−*N*_model_)/(*N*_control_−*N*_model_). The blue dashed line represented the efficacy score of 0.85 and the red dashed line marked the efficacy score of 1.0. Hydroxysafflor yellow A (HSYA) is marked as a green dot and indicated by a black arrow. Compounds that increased the number of HSCs to a higher than normal level are marked as red dots; (E) rescue effects on HSCs with gradient treatment of HSYA in TPEN‐treated zebrafish.

To identify protective compounds against hematopoietic defects associated with P53 hyperactivation, we designed an enriched library consisting of 102 natural molecules. These molecules have previously been implicated in the regulation of blood diseases, either directly (as single molecules) or indirectly (as components of medicinal plants) (Figure 2A). The library comprises 33.60% terpenoids, 20.49% flavonoids, 14.75% phenylpropanoids, 8.20% phenols, 7.38% lignans, 7.38% alkaloids, and 8.2% small molecules from other categories (Figure 2B, Table S1).

Using the number of HSCs in the CHT region as the readout, we screened for molecules with a rescuing effect in TPEN‐treated *Tg(runx1: eGFP)* zebrafish embryos (Figure 2C). An efficacy score was calculated as (*N*_drug_−*N*_model_)/(*N*_control_−*N*_model_), where *N*_drug_ represents the number of HSCs in the drug‐treated group, *N*_model_ represents the number of HSCs in the TPEN‐treated group, and *N*_control_ represents the number of HSCs in the control group. Compounds with an efficacy score higher than 0.85 were considered to have a positive effect on hematopoiesis. Importantly, compounds that increased the number of HSCs to a level higher than that of the normal group were excluded to mitigate the potential risk of hematological malignancies. As a result, a chalcone glycoside, HSYA, was identified as the compound with the most significant hematopoietic‐protective effect (Figure 2D, Table S2). The rescuing effect of HSYA was further validated in a significant dose‐dependent manner in zebrafish (Figure 2E). Consequently, we focused on HSYA for further study.

### 2.3 Regulation of downstream hematopoietic lineages by HSYA

**FIGURE 3:**
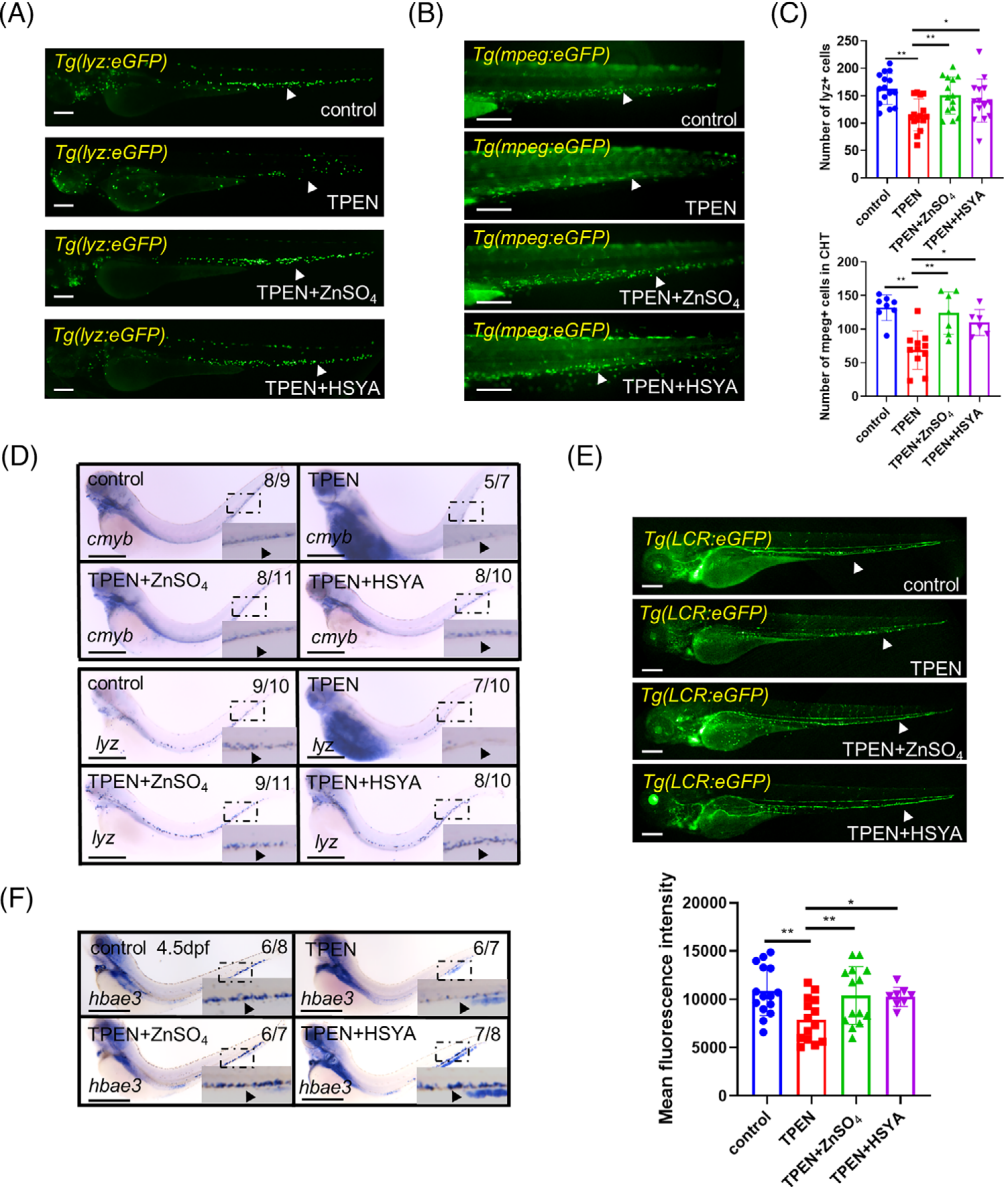
Regulation of downstream hematopoietic lineages by hydroxysafflor yellow A (HSYA) in *N*,*N*,*N*′,*N*′‐tetrakis(2‐pyridylmethyl)ethylenediamine (TPEN)‐treated zebrafish: (A and B) representative images showing neutrophils in *Tg(lyz:eGFP)* embryos (A) and macrophages in *Tg(mpeg:eGFP)* embryos (B) in control and TPEN (150 μM)‐treated embryos with or without incubation of ZnSO_4_ (100 μM) or HSYA (100 μg/ml); (C) quantitative result of neutrophils and macrophages; (D) whole in situ hybridization showing the transcriptional expression of *cmyb* and *lyz* in zebrafish embryos; (E) representative fluorescence images and quantitative results of red blood cells in *Tg(LCR:eGFP)* embryos on 4.5 dpf; (F) whole in situ hybridization showing the transcriptional expression of *hbae3* in zebrafish embryos. The numbers at the top‐right corner of (D–F) show the number of embryos with representative phenotypes/the total number of embryos in the group. Positive stained cells are pointed by a black arrowhead in the zoomed insets of (D) and (F). At least 7 embryos were included in each group; Scale bar (in A, B, and E), 200 μm; scale bar (in D and F), 500 μm. hpf, hours post fertilization. *p < 0.05; **p < 0.01.

We proceeded to analyze the endogenous effects of HSYA on the differentiation of downstream blood lineages in embryos with hematopoiesis defects. As anticipated, the reductions in both neutrophils and macrophages caused by TPEN treatment were effectively counteracted by the HSYA treatment (Figure 3A–C). Furthermore, HSYA treatment successfully restored the circulation of erythrocytes at 4.5 dpf in embryos subjected to 48 h of TPEN treatment (Figure 3E).

To validate the hematopoietic‐protective effects of HSYA at the molecular level, we further examined the in vivo expression of key markers: *cmyb* for HSCs, *lyz* for neutrophils, and *hbae3* for erythroid cells in TPEN‐treated embryos, both with or without HSYA treatment. TPEN significantly downregulated the expressions of these genes, which were largely recovered by HSYA treatment (Figure 3D,F). These findings clearly demonstrate that HSYA rescues the differentiation of multi‐lineage blood progenitor cells in TPEN‐treated zebrafish.

### 2.4 HSYA downregulated P53 expression and cell apoptosis signaling

**FIGURE 4:**
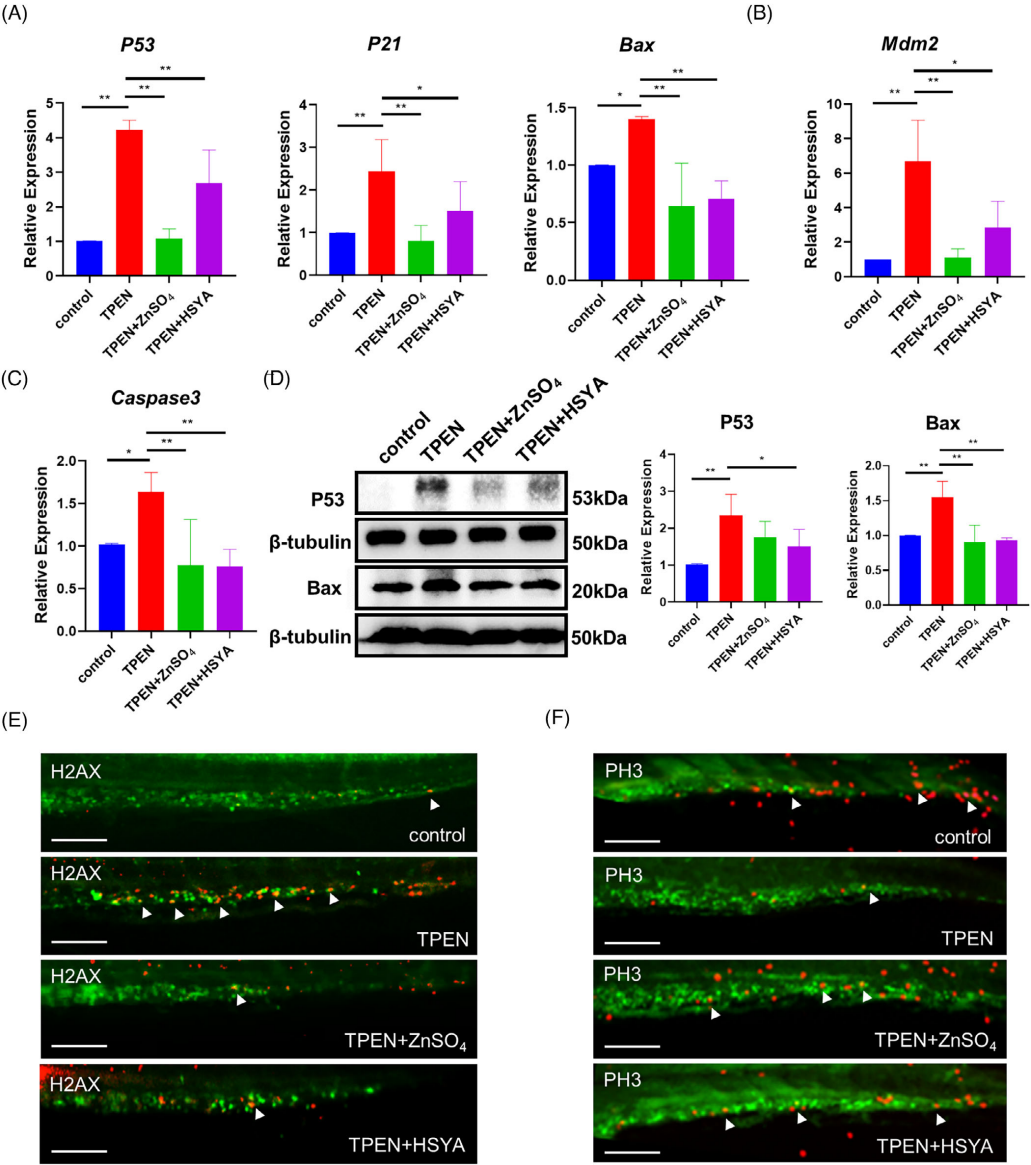
Hydroxysafflor yellow A (HSYA) downregulated P53 signaling and cell apoptosis in *N*,*N*,*N*′,*N*′‐tetrakis(2‐pyridylmethyl)ethylenediamine (TPEN)‐treated zebrafish. (A) QPCR results showing the expression of P53 and its downstream proapoptotic factors P21 and Bax in zebrafish embryos with indicated treatment for 12 h; TPEN:150 μM, ZnSO_4_:100 μM, HSYA:100 μg/ml. (B and C) QPCR results showing the expression of Mdm2 (B) and Caspase3 (C); (D) Western blot images and quantitative results showing P53 and Bax expression; (E and F) immunostaining of H2AX‐labeled apoptotic cells (red signals in E) and PH3‐labeled of proliferating cells (red signals in F) in TPEN‐treated *Tg(runx1: eGFP)* zebrafish with hematopoietic stem cells (HSCs) labeled in green; at least 7 embryos were included in each group; For Western blot and QPCR experiments, 30 embryos in each group from 3 separate experiments were used; scale bar (in E and F), 100 μm. *p < 0.05; **p < 0.01.

Given that our results indicate TPEN‐induced hematological defects are P53‐dependent, we sought to investigate whether HSYA regulates P53 signaling. At the transcriptional level, significantly increased levels of *P53* and its downstream proapoptotic factors *Bax* ^29^ were detected after TPEN stimulation, which were effectively inhibited by HSYA or ZnSO_4_ supplementation (Figure 4A). Moreover, TPEN treatment led to increased expression of the cyclin‐dependent kinase inhibitor *P21*, a key factor mediating P53‐induced G1 cell cycle arrest, which was subsequently reduced by HSYA (Figure 4A). Additionally, the expression of *Mdm2*, a negative regulator of *P53*, was upregulated in the TPEN‐treated group but decreased after HSYA or ZnSO_4_ treatment. This observation is consistent with the known negative feedback loop of P53 on Mdm2 expression^30^ (Figure 4B). The apoptosis marker *Caspase3* was also elevated in TPEN‐treated group and downregulated by HSYA (Figure 4C). The downregulation effects of HSYA administration on the endogenous expression of P53 and Bax in TPEN‐treated groups were further verified at the protein level (Figure 4D).

Immunostaining assays revealed that HSYA effectively decreased the signals of H2AX and increased the signals of PH3 in HSCs, suggesting that the molecule can promote the rebalancing of cell apoptosis and proliferation (Figure 4E,F). We further generated a *P53*‐overexpressed zebrafish model by microinjection of *P53* plasmid (Figure S1). As observed in TPEN‐treated embryos, a severe reduction in the number of HSCs was observed. However, HSYA treatment significantly alleviated the hematopoietic defects. Additionally, approximately 10% of embryos with P53 overexpression displayed other abnormal phenotypes, including edema, tail dysplasia, and craniofacial deformities. These severe phenotypes may be a result of early P53 overexpression (from the one‐cell stage) throughout the entire embryo. In summary, these results suggest that HSYA alleviates HSC injury in zinc‐deficient zebrafish, possibly by inhibiting hyperactivated P53 signaling.

### 2.5 Validation of HSYA activity on zinc‐deficient mice bone marrow nucleated cells

**FIGURE 5:**
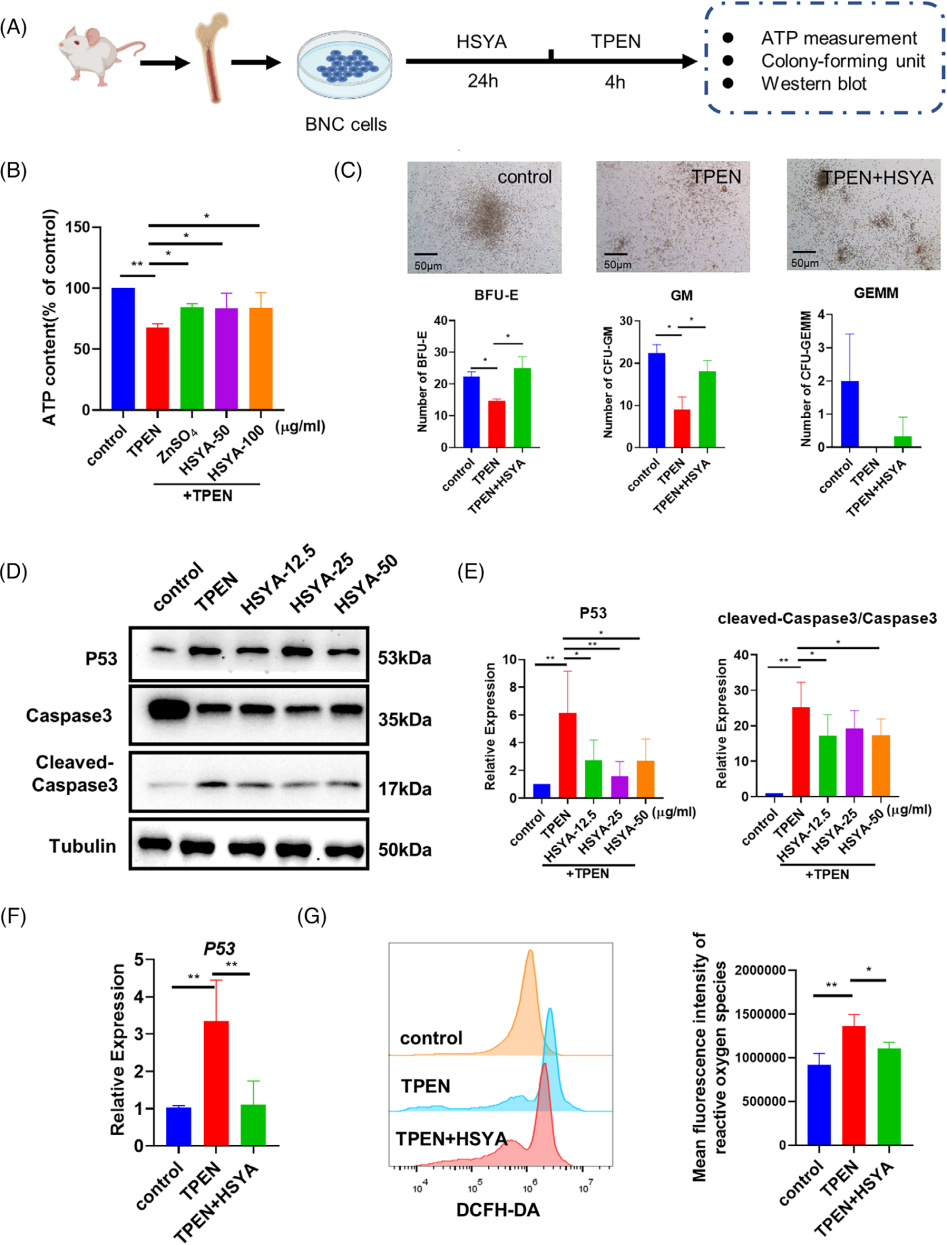
Hematopoietic‐promoting activity of hydroxysafflor yellow A (HSYA) on mice bone marrow nucleated cells: (A) experimental protocol of bone marrow nucleated cells (BNCs) extraction and drug treatment; (B) ATP content of control and *N*,*N*,*N*′,*N*′‐tetrakis(2‐pyridylmethyl)ethylenediamine (TPEN)‐treated BNCs with or without HSYA pretreatment; (C) representative images and quantitative result of hematopoietic colony‐forming unit (CFU) assay. (D and E) Western blot images and quantitative results showing P53 and cleaved‐Caspase3/Caspase3 expression of control and TPEN‐treated BNCs with or without different dosages of HSYA; (F) QPCR results showing the expression of P53 in BNCs with indicated treatment; (G) fluorescence intensity and quantitative result of reactive oxygen species detected by flow cytometry. The dosage of TPEN is 7.5 μM in all groups; the dosages of HSYA are 50 μg/ml in (C) and 12.5 μg/ml in (F and G), and as listed in (B) and (D). BFU‐E, burst‐forming unit‐erythroid; GM, granulocyte–monocyte; GEMM, granulocyte‐erythrocyte‐macrophage‐megakaryocyte. Scale bar in (C), 50 μm; *p < 0.05; **p < 0.01.

We proceeded to investigate whether the hematopoietic‐protective effects of HSYA observed in zebrafish was also applicable to mammalian cells. Bone marrow nucleated cells (BNCs) from mice were collected and cultured in TPEN‐supplemented medium to create a low‐zinc microenvironment (Figure 5A). The ATP activity assay indicated a moderate reduction in cellular viability of BNCs under zinc deficiency. However, zinc or HSYA administration effectively elevated ATP content, indicating improved cellular viability (Figure 5B). The downstream differentiation capability of mice HSCs into different blood lineages was examined by hematopoietic colony‐forming unit (CFU) assay. Severe impairment of HSCs differentiation was observed after TPEN treatment, with substantial reductions in the colony numbers of both burst‐forming unit‐erythroid (BFU‐E) and granulocyte–monocyte (GM). Remarkably, HSYA treatment significantly restored the downstream differentiation of zinc‐deficient HSCs (Figure 5C). Although the trend of GEMM quantities also aligned with that of BFU‐E and GM, statistical significance was not observed.

Consistent with the findings in zebrafish, the protein expressions of P53 and cleaved‐Caspase3 increased after TPEN treatment but were significantly downregulated by HSYA (Figure 5D,E). Additionally, the P53 inhibitor pifithrin‐α largely blocked TPEN‐induced cell death in mice BNCs (Figure S2). Next, we investigated how HSYA regulates P53 expression. Our previous studies have shown increased protein stability of P53 in zinc‐deficient models.^11^ Thus, we tested if HSYA counteracted TPEN‐induced hematological defects by accelerating P53 degradation. Interestingly, our assay revealed that a higher amount of P53 protein was observed in mice BNCs co‐treated with TPEN and the protein synthesis inhibitor cycloheximide (CHX), compared to CHX‐treated control BNCs, suggesting that P53 protein is more stable in a zinc‐deficient environment. However, our assay did not show a significant impact of HSYA on the P53 protein stability (Figure S3). Nevertheless, similar to the findings in zebrafish (Figure 4A), we detected a robust increase of *P53* mRNA level in TPEN‐treated mice BNCs, which was downregulated by HSYA. This suggests that HSYA could possibly have a negative impact on *P53* transcription (Figure 5F). In addition, oxidative stress has been established as an essential inducer of P53 expression and hematopoietic homeostasis.^31^, ^32^ Excessive ROS accumulation, as indicated by the DCFH‐DA probe, was detected in BNCs, and HSYA treatment significantly downregulated the ROS level (Figure 5G). Moreover, *N*‐acetyl‐l‐cysteine (NAC), an ROS inhibitor, increased cell viability and decreased the level of P53 transcription in TPEN‐treated mice BNCs (Figure S4). Therefore, the above results suggest that the protective effects of HSYA on hematopoiesis are conserved in mammalian cells, potentially related to its negative regulation on P53 transcription and oxidative stress.

### 2.6 HSYA partially rescued hematopoietic defects in 5‐FU‐treated mice

**FIGURE 6:**
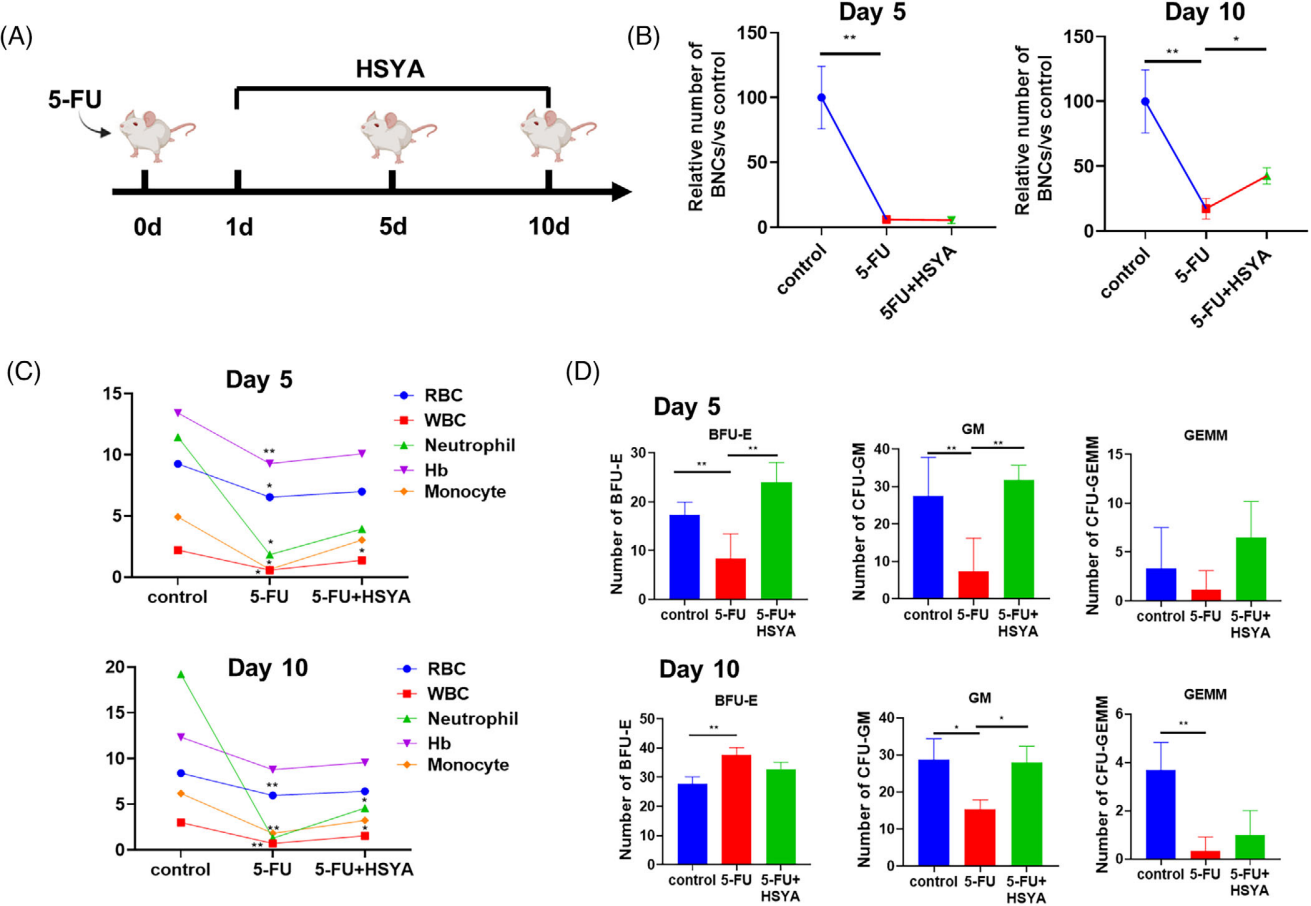
Hydroxysafflor yellow A (HSYA) partially rescues hematopoietic defects in 5‐FU‐induced myelosuppression mice: (A) timeline of 5‐FU modeling and drug administration; (B) the number of bone marrow nucleated cells (BNCs) in the 5‐FU‐treated mice with or without HSYA administration on days 5 and 10. Each group had five mice; (C) peripheral blood analysis on days 5 and 10. Each group consisted of 3–5 mice; (D) quantitative result of hematopoietic colony‐forming unit (CFU) assay. *p < 0.05; **p < 0.01.

The protective effect of HSYA on hematopoietic injury was further examined in a 5‐FU‐stimulated hematopoietic disorder model in mice, as multiple studies have attributed 5‐FU‐induced bone marrow toxicity to P53 hyperactivation.^33^, ^34^ To evaluate the impact of 5‐FU on mouse hematopoietic function, two observation time points were selected: day 5 (acute injury stage) and day 10 (recovery stage) after 5‐FU injection (Figure 6A). The number of BNCs in the 5‐FU treatment group dramatically decreased on both days 5 and 10, with mild recovery observed at the latter time point. Although HSYA administration failed to increase the number of BNCs on day 5, it significantly increased BNCs on day 10 (Figure 6B). Besides, peripheral blood analysis detected severe reductions in the numbers of erythrocytes and leukocytes in 5‐FU‐stimulated mice on both days 5 and 10. However, HSYA administration partially rescued the numbers of leukocytes and neutrophils (Figure 6C). As suggested by the CFU assay, the differentiation of GM and GEMM was largely impaired after 5‐FU exposure. Interestingly, the number of BFU‐E colonies increased to an even higher level than the control group on day 10 (Figure 6D). Noticeably, HSYA administration effectively restored the number of CFUs at both observation time points.

**FIGURE 7:**
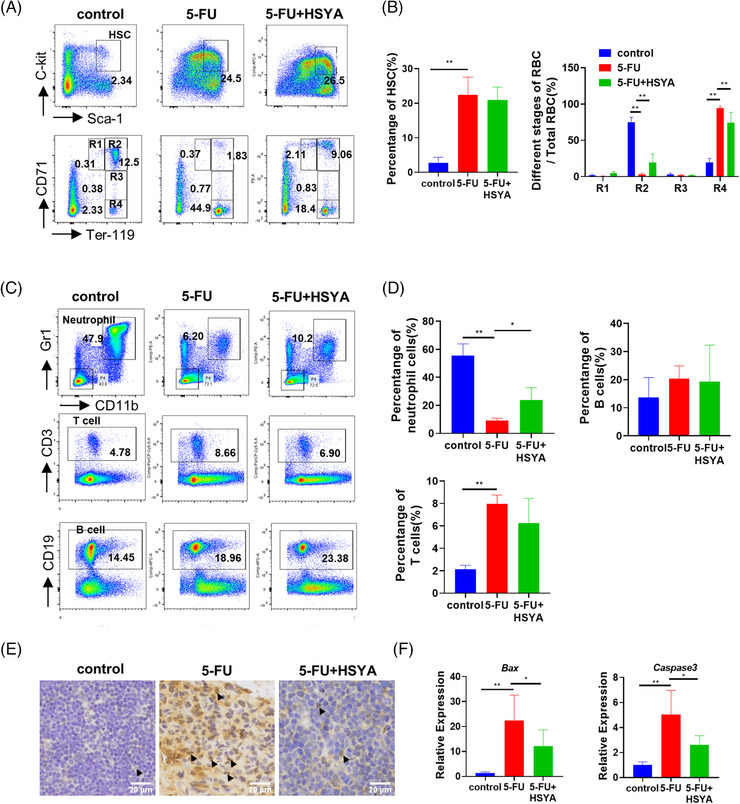
Hydroxysafflor yellow A (HSYA) partially repairs hematopoietic defects in 5‐FU‐treated mice: (A) flow cytometry in different treatment groups to detect the proportion of hematopoietic stem cells and the distribution of erythrocytes at different stages on day 10. LSK cells/hematopoietic stem cells (HSCs) (Lin‐Sca‐1^+^c‐kit^+^); proerythroblast/R1 (CD71^high^Ter119^intermediate^); basophilic erythroblast (CD71^high^Ter119^high^); polychromatic erythroblast (CD71^intermediate^Ter119^high^); orthochromatic erythroblast (CD71^low^Ter119^high^); 5‐FU, 200 mg/kg; HSYA, 100 mg/kg; (B) quantitative result of flow cytometry; (C) flow cytometry to detect the proportion of neutrophils, T cells and B cells on day 10. Neutrophil (CD11b^+^Gr1^+^); T lymphocyte (CD3^+^); B lymphocyte (CD19^+^); (D) quantitative result of (C) for flow cytometry; three mice in each group for flow cytometry; (E) immunohistochemical staining of P53 in mouse thymus tissue. Arrowheads pointed to P53‐stained cells. Scale bar: 20 μm; (F) QPCR results showing the expression of Bax and Caspase3 in different mice treatment groups. *p < 0.05; **p < 0.01.

The numbers of HSCs and downstream lineages were further examined in the mice bone marrow by FACS on day 10 after 5‐FU stimulation. A significant expansion of LSK cells (Lin^−^Sca‐1^+^c‐kit^+^) was observed in the 5‐FU‐treated group, possibly as a compensatory amplification of HSCs after injury (Figure 7A,B). The distribution pattern of erythrocytes at different stages was greatly skewed after 5‐FU stimulation, with a substantial reduction in early erythroblasts (CD71^high^Ter119^high^) and a significant increase in the proportion of reticulocytes/mature erythroblasts (CD71^low^Ter119^high^) (Figure 7A,B). HSYA administration alleviated the imbalanced differentiation of erythrocytes. Moreover, leukocyte analysis detected a reduced number of neutrophils (CD11b^+^Gr1^+^), accompanied by an increased number of T lymphocytes (CD3^+^). HSYA supplementation moderately upregulated the number of neutrophils (Figure 7C,D). In addition, histochemistry staining showed a significant increase in P53 staining in the thymus of TPEN‐treated mice, which was effectively reduced by HSYA. Enhanced expressions of *Bax* and *Caspase3* were also detected in the bone marrow of 5‐FU‐treated mice, and this expression was downregulated by HSYA (Figure 7E,F).

Finally, to evaluate whether HSYA has a potential value in the prevention of 5‐FU‐induced hematopoietic toxicity without affecting its chemotherapeutic effects, the potential impact of HSYA on the antitumor efficacy of 5‐FU was evaluated using four tumor cell lines: hepatocellular carcinoma cells (HepG2), breast cancer cells (MDA‐MB‐436), colon cancer cells (Mc38), and cervical cancer cells (HeLa). No interfering effect was detected for HSYA regarding the tumor cell toxicity of 5‐FU (Figure S5). In summary, the above results suggest that HSYA attenuates 5‐FU‐induced bone marrow damage without affecting the toxic effects of 5‐FU on tumor cells.

## 3 DISCUSSION

Hematopoietic homeostasis is tightly regulated by multiple signaling pathways. Recent studies in the elderly individuals and patients undergoing chemotherapy have indicated that P53 hyperactivation is a major cause of hematopoietic defects.^6^, ^35^ In this study, we identified the novel hematopoietic‐protective role of a water‐soluble chalcone glycoside HSYA in zebrafish and mouse models of hematopoietic disorders associated with P53 overexpression.

To mimic P53 hyperactivation‐associated hematopoietic defects, we utilized two modeling strategies. Inadequate zinc intake is a common form of malnutrition among the elderly population.^36^ Clinical studies have shown that reduced serum zinc levels are recognized as unfavorable prognostic factors in patients with aplastic anemia, iron deficiency anemia, and leukemia.^37^, ^38^, ^39^ In mice supplemented with a zinc‐deficient diet for 34 or 50 days, increased apoptosis was observed in both erythroid and lymphoid lineages, along with thymus atrophy, indicating disrupted hematopoiesis under limited zinc supply.^15^, ^26^ Although our studies and those of other groups have established the fundamental role of P53 overactivation in zinc deficiency‐induced cell deaths in macrophages and neuronal precursor cells,^28^, ^40^, ^41^, ^42^ the relationship between P53 signaling and zinc deficiency‐induced hematopoietic malfunctions remains unclear. In our study, treatment with TPEN led to severe reduction of HSCs and multi‐lineage hematopoietic defects in zebrafish, which were completely blocked by the usage of *tp53*‐translational blocking morpholino. The P53 inhibitor pifithrin‐α also largely prevented cell toxicity in TPEN‐treated BNCs. Although diverse biological factors and pathways could be involved in zinc deficiency and collectively cause hematopoietic dysfunction, our findings support the indispensable role of the P53 signaling pathway in this pathological process. Interestingly, in our recent study on zinc importer *Slc39a10* knockout mice, although increased P53 signaling was also detected, P53 inhibition alone was insufficient to restore the survival of *Slc39a10*‐deficient HSCs.^43^ One possible explanation for this inconsistency is that other divalent metal ions transported by the Slc39a10 protein, such as iron, manganese, and cadmium, may exert P53‐independent roles in hematopoiesis.^44^

Chalcone, as a precursor for flavonoids and isoflavonoids, serves as a common scaffold for many naturally occurring compounds. Chalcone derivatives are considered privileged structure in medicinal chemistry due to their convenient synthesis.^45^ As one of these derivatives, the natural chalcone glycoside HSYA has been associated with the treatment of a wide range of diseases, particularly cardiovascular diseases. In our study, we identified the hematopoietic‐protective activity of HSYA for the first time. Based on the results from both zebrafish and mice models, we speculate that the protective effects of HSYA on hematopoiesis are at least partially related to the P53 signaling. P53 hyperactivation is known to negatively regulate HSC pool size by inducing cell apoptosis and cell cycle arrest.^46^ Consistently, we observed increased apoptotic signal and decreased cell proliferation in the HSCs of TPEN‐treated zebrafish with overexpressed P53 signaling. However, HSYA treatment was able to rescue these defects. At the molecular level, we found that HSYA decreased the expression of P53 and the proapoptotic factors Bax and Caspase3 and also inhibit the excessive expression of P21, which is a cyclin‐dependent kinase inhibitor involved in cell cycle arrest.^47^, ^48^ Nevertheless, it is also possible that HSYA has P53‐independent roles in hematopoiesis. For example, previous studies suggested that HSYA could increase the nuclear accumulation of β‐catenin^49^ and inhibit the activation of NLRP3 inflammasome,^50^ both of which play pivotal roles in hematopoiesis physiology.^51^, ^52^ Further studies are warranted to elucidate the molecular mechanism of HSYA in the hematopoietic process.

Our study revealed the negative regulation of HSYA on P53 expression. This finding is consistent with previous reports showing that HSYA protects human umbilical vein endothelial cells from hypoxia‐induced cell apoptosis and cell cycle arrest by downregulating P53 and the ratio of bax/bcl‐2.^53^ In addition, HSYA has been found to inhibit the high expression of P53 to reduce apoptosis in a rat model of diabetic retinopathy.^54^ However, the regulatory mechanism of HSYA on P53 signaling remains unclear, and we propose several possibilities based on our findings. First, as observed in both zebrafish and mice bone marrow cell models, HSYA downregulates the transcriptional level of P53. Therefore, one possibility is that HSYA may interact with certain transcription factors in the cytoplasm, which subsequently translocate into the nucleus to regulate P53 transcription. Second, the strong anti‐oxidative properties of HSYA could potentially contribute to its impacts on P53 expression. Accumulation of ROS in HSCs is known to activate the P53 pathway, presumably in response to DNA damage.^31^ Consistently, coordinated alterations in ROS level and P53 activation were observed in the mice BNCs in our study, as well as in several other findings.^55^, ^56^ We also detected a negative modulation of the ROS inhibitor NAC on the level of P53 expression in TPEN‐treated mice BNCs. However, the crosstalk between oxidative stress and P53 signaling is complex, and whether the impact of HSYA on P53 expression is mediated by ROS remains unclear. Future studies investigating the protein interaction of HSYA by approaches such as biotinylated small molecule pull‐down assay will be valuable in further elucidating the regulatory mechanism of HSYA on the P53 signaling pathway.

Aside from zinc deficiency, chemotherapy‐induced bone marrow suppression is another common cause of P53 overexpression‐related hematopoietic defects. Bone marrow toxicity is a severe side effect of many chemotherapeutic and DNA damaging drugs, such as Adriamycin, 5‐FU, and cisplatin, all of which are associated with increased P53 activation. In this study, we used a 5‐FU stimulated mouse model to verify the hematopoietic‐protective effects of HYSA in mammals. Multiple mechanisms have been proposed for 5‐FU‐induced P53 activation, including an incorporation of fluorouridine triphosphate or fluorodeoxyuridine triphosphate into DNA or RNA, and the inhibition of thymidylate synthase.^57^ Consistent with previous studies,^58^, ^59^ we detected time‐dependent alterations in hematopoietic profiles after 5‐FU administration, with significant inhibition in the early stage and gradual recovery in the later stage. Interestingly, although reductions in all‐lineage blood cells were observed in the peripheral blood of 5‐FU‐treated mice, the recovery of erythroid lineages was faster than that of myeloid cells and lymphocytes, as indicated by the CFU assay and bone marrow cell FACS. This could be attributed to the rapid compensatory amplification of erythroid cells in response to hypoxia stress. HYSA significantly promoted hematopoietic recovery in 5‐FU‐treated mice, particularly in the myeloid lineages. Furthermore, HSYA had no effect on the chemotoxicity of 5‐FU in multiple tumor cell lines, supporting its potential application in cancer patients experiencing bone marrow suppression due to 5‐FU chemotherapy. Importantly, recent studies have suggested that, in addition to its role in HSC depletion, gain‐of‐function mutant P53 proteins have oncogenic effects on hematopoiesis, which may contribute to clonal hematopoiesis, myeloid leukemia, and other hematological malignancies.^60^, ^61^, ^62^ Although excessive proliferation of hematopoietic cells was not observed in our models, it is clinically significant to investigate the potential therapeutic effects of HSYA on P53 activation‐related hematological malignancies in future studies.

Finally, our study has several limitations that should be addressed. First, although we propose that hyperactivated P53‐induced apoptosis plays an essential role in hematopoietic malfunctions, other cell death signaling pathways, such as necroptosis^63^ and ferroptosis,^64^ may also contribute to this complex pathological process. Exploring the interplay among different forms of cell death in hematopoietic disorders is an interesting avenue for future investigation. Second, although our work demonstrates the strength of multi‐model‐based drug screening in identifying HSYA as a novel hematopoietic‐protective molecule, the in‐depth mechanism of HSYA on P53 signaling and hematopoiesis remains unclear. Further studies are warranted to identify the protein target of HSYA that directly mediates its protective effects against P53 overactivation‐associated hematopoietic defects. Lastly, our study only tested the effects of HSYA in zebrafish and mouse models. It would be highly valuable to include clinical samples from hematopoietic deficient patients, particularly zinc‐deficient elderly individuals and patients with chemotherapy‐related bone marrow suppression, to further evaluate the potential therapeutic effects of HSYA.

In conclusion, our study highlights the indispensable role of P53 in zinc deficiency‐related hematopoietic defects and presents a convenient phenotypic screening strategy for identifying hematopoietic‐protective compounds. Through this strategy, we demonstrated the potent effects of HSYA, a natural chalcone derivative, in maintaining the cell survival and differentiation of HSCs and multiple blood progenitor cells in both zebrafish and mouse models, as well as its strong inhibition of the P53 signaling pathway and oxidative stress. HSYA shows promise as a lead compound for the treatment of hematopoietic disorders.

## 4 MATERIALS AND METHODS

### 4.1 Chemicals and reagents

TPEN (16858‐02‐9, Sigma‐Aldrich), ZnSO_4_ (204986, Sigma‐Aldrich), anti‐phospho‐histone H3 (Ser10) antibody (06‐570, Sigma‐Aldrich), HSYA (B20968, Yuanye), which purity was greater than 98% (HPLC), anti‐GFP antibody (11814460001, Roche), anti‐p53 antibody for zebrafish (GTX128135, GeneTex) UltraSYBR Mixture (CW0957, Cwbio), HiFiScript cDNA Synthesis Kit (CW2569, Cwbio), RNA‐Quick Purification Kit (ES‐RN001, Yeasen), Anti‐Phospho‐Histone H2A.X (Ser139) antibody (9718, CST), anti‐P53 (1C12) antibody for mouse (2524, CST), anti‐Bax antibody (89477, CST), anti‐Caspase3 antibody (14220, CST), anti‐cleaved Caspase3 antibody (9664, CST), CellTiter‐Glo Luminescent Cell Viability Assay (G7570, Promega), Mouse CFU Assays Using MethoCult (M3434, Stemcell), CHX (NSC‐185, Selleck), 5‐Fluorouracil (F100149, Aladdin), ROS assay kit (S0033, Beyotime), Flow cytometry antibody Alexa Fluor 700 anti‐mouse Lineage Cocktail with Isotype Ctrl (133313), APC anti‐mouse CD117 (c‐kit) (105811), PE/Cyanine7 anti‐mouse Ly‐6A/E (Sca‐1) (108112), APC anti‐mouse TER‐119/erythroid Cells (116211), PE anti‐mouse CD71 (113807), APC/Cyanine7 anti‐mouse CD11b (101225), PE anti‐mouse Ly‐6G/Ly‐6C (Gr‐1) (108407), PerCP/Cyanine5.5 anti‐mouse CD3ε (100327), and APC anti‐mouse CD19 (152409) were purchased from BioLegend.

### 4.2 Zebrafish husbandry

Transgenic fish lines, including HSCs‐labeled Tg*(runx1: eGFP)*, neutrophils‐labeled Tg*(lyz:eGFP)*, macrophages‐labeled Tg*(mpeg:eGFP)*, and erythrocytes‐labeled Tg*(LCR:eGFP)*, were previously generated.^65^, ^66^, ^67^, ^68^ All zebrafish lines were obtained from the Laboratory Animal Center of Zhejiang University and were kept and maintained according to standard protocols. Zebrafish embryos were cultured in E3 medium (0.29 g/L NaCl, 0.013 g/L KCl, 0.048 g/L CaCl_2_·2H_2_O, 0.082 g/L MgCl_2_·6H_2_O, pH 7.2). A 0.3 M tricaine solution was used as an anesthetic.

### 4.3 Phenotypic screening on TPEN‐induced hematopoietic disorder in zebrafish

HSCs‐labeled *Tg(runx1: eGFP)* embryos were treated with 150 μM TPEN at 2.5 dpf to induce the hematopoietic disorder model. For drug screening, natural compounds (100 μM) or ZnSO_4_ (positive drug, 100 μM) were added simultaneously with TPEN to evaluate the protective effects, with 5–8 embryos per group. The dosages of TPEN and ZnSO_4_ were determined based on our previous experiences and other studies.^43^ DMSO was used as the solvent for compounds, and the control group was treated with 0.1% DMSO. The embryos were photographed with Leica DMI 3000B inversed microscope system (Leica Microsystems) at 12 h after treatment, and the number of HSCs in the CHT region was counted using ImageJ (1.53c) according to the following steps: Process‐Filters‐Convolve; Process‐Find Maxima (Noise tolerance changed to 30)‐Count.

### 4.4 Whole embryo in situ hybridization

Briefly, embryos were fixed in 4% formalin in PBS and incubated with digoxigenin‐UTP labeled probes. An alkaline phosphatase‐coupled anti‐digoxigenin antibody (11093274910, Roche, 1:1000 dilution) was used to localize hybridized probes, and NBT (Nitro Blue Tetrazolium) (11383213001, Roche)/BCIP (5‐bromo‐4‐chloro‐3‐indolylphosphate) (11383221001, Roche) was used as the chromogenic substrate to produce blue precipitates. Whole‐mount in situ hybridization was performed as described before.^69^, ^70^

### 4.5 Western blot

For mouse BNCs, 2 × 10^6^ cells were collected per sample; for zebrafish, 30 embryos were collected per group. Samples were lysed on ice for 10 min in lysis buffer containing 1% phenylmethanesulfonyl fluoride and 1% protease inhibitor cocktail and then centrifuged at 12,000 rpm for 10 min at 4°C. For each sample group, 10 μg protein of cell and 30 μg of zebrafish were used for Western blot analysis. The antibody was diluted at 1:1000. The expression of β‐tubulin was used as the loading control. Images of blots were obtained by using Chemidoc Imaging System (Bio‐Rad).

### 4.6 CHX treatment

CHX (25 μg/m) was used to inhibit protein synthesis and evaluate the stability of protein.^11^ HSYA was added after BNCs were extracted and pretreated for 24 h for HSYA administration group. Then BNCs were cultured in the absence or presence of TPEN (7.5 μM), with or without CHX treatment for 3 h. After drug treatment, cellular proteins were extracted for Western blot assay.

### 4.7 Zebrafish microinjection

The translation of P53 was inhibited by 1.76 ng/embryo morpholino injection (*tp53* MO: 5′‐GCG CCA TTG CTT TGC AAG AAT TG‐3′, Gene Tools) at one‐cell stage. Injection of standard control morpholino (control MO: 5′‐CCT CTT ACC TCA GTT ACA ATT TAT A‐3′, Gene Tools) of the same dosage was used as a negative control.

### 4.8 Immunofluorescent staining

Embryos were fixed overnight in 4% paraformaldehyde at 4°C, digested with proteinase K (10 μg/ml) for 30 min and penetrated with precooled acetone at −20°C for 7 min. Anti‐GFP (1:500), anti‐PH3 (1:1000), or anti‐H2A.X (1:300) were used as first antibodies for overnight incubation. Fluorescent secondary antibodies were diluted to 1:400 and incubated for 2 h at 4°C. Embryos were mounted with Fluoro‐Gel mounting medium (17985‐10, Electron Microscopy Science) and analyzed using the Leica DMI 3000B inversed microscope system (Leica Microsystems).

### 4.9 Quantitative PCR

Total RNA was extracted from 30 zebrafish embryos with an RNA‐Quick Purification Kit for each sample group. The RNA was then reverse‐transcribed to single strand cDNA with HiFiScript cDNA Synthesis Kit. Real‐time PCR was performed using the two‐step quantitative RT‐PCR method with 2X SYBR Green qPCR Mater Mix. Ef‐1α was used as the internal control. The sequences of all primers used in the study are listed in Table S3.

### 4.10 Extraction of mouse bone marrow nucleated cells

The experimental mice were male C57BL/6 J mice, 6–8 weeks old, purchased from SLAC. Mouse BNCs were collected from the bone marrow cavity of mice's femur using RPMI‐1640 medium. The cell suspension was passed through a 100 μm cell sieve, transferred to a centrifuge tube, and centrifuged at 4°C for 5 min at 1500 rpm. Then, 2 mL of erythrocyte lysate was added to the cell pellet, followed by the addition of 10 mL culture medium to terminate erythrocyte lysis. The cells were centrifuged at 4°C for 5 min at 1500 rpm. Finally, the cells were resuspended in culture medium and seeded into 96‐well cell culture plates at a density of 3 × 10^4^/well.

### 4.11 Assaying of ATP content

CellTiter‐Glo Reagent was added to BNCs, and the cells were lysed by shaking for 2 min, followed by 10 min at room temperature to stabilize the luminescent signal. The bioluminescence values were measured using an Infinite F200 multifunctional enzyme marker for 1 s. The mean values were calculated for each drug concentration in triplicate wells. The ATP content was calculated according to the following formula.

ATP content (%) = bioluminescence value of sample group/bioluminescence value of control group × 100%.

### 4.12 Mouse colony‐forming unit (CFU) assays

Hematopoietic progenitor cell colony generation assays were performed according to the manufacturer's instructions using MethoCult. The concentration of BNCs was adjusted to 2 × 10^5^/mL. A 0.3 mL cell suspension was added to 3 mL of methylcellulose medium, vortexed, mixed, and left for 5 min. Then, 1.1 mL of the culture medium with or without TPEN (7.5 μM) was added to a 35 mm covered culture dish, creating two replicate wells. The cells were incubated in a 37°C cell culture incubator for 10–12 days and examined under a microscope. The types of CFUs were determined based on their distinctive morphologies as guided in MethoCult.

### 4.13 Assaying of ROS

Cells were collected, centrifuged at 500 *g* for 5 min, and washed three times with PBS to remove residual medium. A dilution of 10 μM ROS staining probe DCFH‐DA (S0033S, Beyotime) was added, and the cells were incubated at 37° for 20 min. After staining, the cells were washed three times with PBS, filtered through a cell sieve, and then fluorescence intensity was detected by flow cytometry.

### 4.14 In vivo experiments on mice

C57BL/6 J mice (male, 6–8 weeks, SLAC) were used for the experiment. Prior to blood and tissue collection, mice were anesthetized by intraperitoneal injection of 50 mg/kg of sodium pentobarbital at a dose of 0.1 mL/20 g according to the body weight of the mice. The mice were randomly divided into three groups: the blank control group (control), the 5‐FU group (model), and the HSYA administration group (5‐FU + HSYA). The model group and the drug administration group received a single intraperitoneal injection of 200 mg/kg 5‐FU to construct a myelosuppression model, whereas the control group received equal amounts of saline intraperitoneal. The day of modeling was recorded as day 0. Starting from day 1, HSYA (100 mg/kg) was administered intraperitoneally for 4 or 9 days for the drug administration groups, and the blood and bone marrow cells were collected on day 5 (acute injury period) and day 10 (hematopoietic repair period) for subsequent analysis.

### 4.15 Flow cytometry analysis

BNCs were adjusted to a concentration of 1 × 10^7^/mL and transferred to 1.5 mL centrifuge tubes, with 100 μL in each tube. Two tubes were prepared, one for HSC flow analysis and the other for neutrophil and lymphocyte flow analysis. An additional batch of BNCs without red cleavage was prepared for erythrocyte flow analysis. After antibody incubation, 1 mL of PBS was added, and the cells were centrifuged at 300*g* for 5 min. The cells were then resuspended in 300 μL PBS and passed through a 300‐mesh cell sieve for flow cytometry analysis.

### 4.16 P53 overexpression in HSCs‐labeled zebrafish embryos

The coding sequence region of the p53 gene was amplified using zebrafish cDNA as a template, with a 6 × His sequence inserted at the 3′ end. The PCR product was ligated to a PCS2 vector by homologous recombination. P53 plasmids (0.2 ng/embryo) were microinjected at the one‐cell stage zebrafish embryos. HSYA (100 μg/mL) was administered at 1 dpf, and the fluorescence images were taken on 3 dpf.

### 4.17 NAC treatment

NAC (S1623, Selleck) at a concentration of 5 mM^71^ was added to mice BNCs 24 h before and immediately after the treatment of TPEN (7.5 μM). ATP content was measured after 4 h, and cellular RNA and protein were extracted for qPCR and Western blot assays, respectively.

### 4.18 TUNEL staining

The P53 inhibitor pifithrin‐α (S1816, Beyotime) at a concentration of 1 μM^72^ and TPEN (7.5 μM) were added together to mice BNCs for 4 h and detected by the TUNEL assay (C1086, Beyotime). For the TUNEL assay, cells were collected and fixed in 4% paraformaldehyde for 30 min, permeabilized with 0.3% Triton X‐100 for 5 min, and incubated with TUNEL assay solution for 1 h in the dark at 37°C.

### 4.19 Statistical analysis

All data are presented as the mean ± the standard error of the mean. At least three independent replications were carried out for each assay for statistical analysis. Differences between two groups were analyzed using the two‐tailed Student's *t*‐test. Multiple group comparison was conducted by one‐way ANOVA with the Dunnett post hoc test. A *p* value <0.05 was considered as statistically significant. GraphPad Prism (8.0.2.263) was used to perform all the statistical analysis.

## AUTHOR CONTRIBUTIONS

*Conception and design*: Lu Zhao, Yi Wang, and Mirko Baruscotti. *Acquisition of data*: Jing Chen, Can Ren, and Chong Yao. *Analysis and interpretation of data*: Jing Chen. *Writing of the manuscript*: Lu Zhao, Yi Wang, and Jing Chen. All authors reviewed and revised the final version of this manuscript and approved its submission.

## CONFLICT OF INTEREST STATEMENT

The authors declare no conflicts of interest.

## ETHICS STATEMENT

All animal experiments were conducted according to the guidelines of the Animal Ethics Committee of the Laboratory Animal Center, Zhejiang University (No. ZJU20230216).

## Supporting information

Supporting Information

## Data Availability

The data that support the findings of this study are available from the corresponding author upon reasonable request.
