# Supplementary material for: Identification of the natural chalcone glycoside hydroxysafflor yellow A as a suppressor of P53 overactivation‐associated hematopoietic defects
Source: MedComm (2020). 2023 Aug 24;4(5):e352. doi: 10.1002/mco2.352 (PMC10449056; doi:10.1002/mco2.352)
Supplement: Supplementary file 1 — Supporting Information [file MCO2-4-e352-s001.docx]

**Identification of the natural chalcone glycoside hydroxysafflor yellow A as a suppressor of P53 overactivation-associated hematopoietic defects**

**Jing Chen^1^, Can Ren^1^,** **Chong Yao^2^, Mirko Baruscotti^3^, Yi Wang^1,4,5^, Lu Zhao^1*^**

^1^Pharmaceutical Informatics Institute, College of Pharmaceutical Sciences, Zhejiang University, Hangzhou 310012, China

^2^Huzhou Central Hospital, Affiliated Huzhou Hospital, Zhejiang University School of Medicine,

Huzhou 313000, China

^3^ Department of Biosciences, University of Milano, Milan 1-20133, Italy

^4^ Innovation Institute for Artificial Intelligence in Medicine of Zhejiang University, Hangzhou 310020, China

^5^ National Key Laboratory of Chinese Medicine Modernization, Innovation Center of Yangtze River Delta, Zhejiang University, 314100, Jiaxing, China.

* Corresponding author.

Lu Zhao: Pharmaceutical Informatics Institute, College of Pharmaceutical Sciences, Zhejiang University, Hangzhou 310012, China

E-mail address: lzhao@zju.edu.cn.

**Supplementary Figures S1-S5**


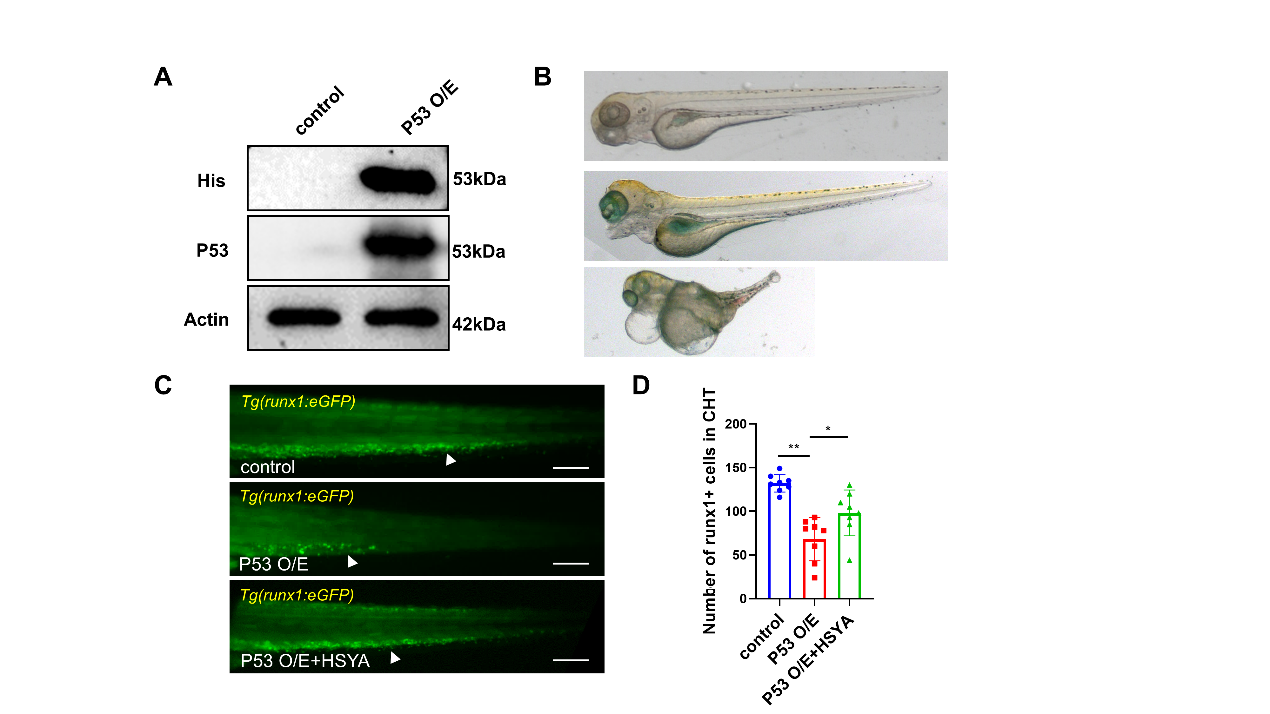


**Figure S1:** HSYA rescued the number of hematopoietic stem cells in P53-overexpressed zebrafish embryos. (A) Western blot showing the expression of His tag and P53 in zebrafish lysate. (B) Representative images of severe abnormal phenotypes of P53-overexpressed zebrafish embryo. Top: control embryo; Middle and bottom: P53-overexpressed embryos with craniofacial deformity (middle), edema and tail dysplasia (bottom). (C-D) Representative images and quantitative results of HSCs number. Scale bar: 200 µm. *, *P* < 0.05; **, *P* < 0.01.


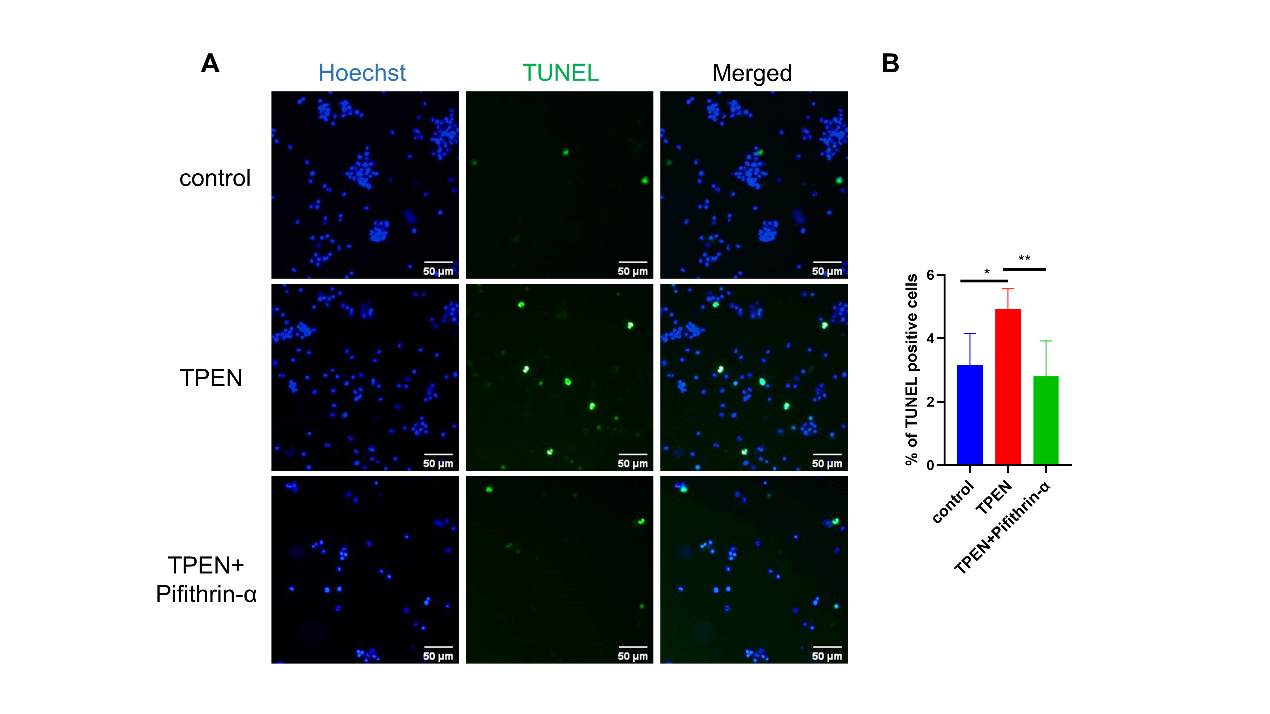


**Figure S2:** P53 inhibitor pifithrin-α reduced cell death in TPEN-treated mice bone marrow nucleated cells. (A-B) Representative images and quantitative results of TUNEL staining. Scale bar: 50 µm. *, *P* < 0.05; **, *P* < 0.01.


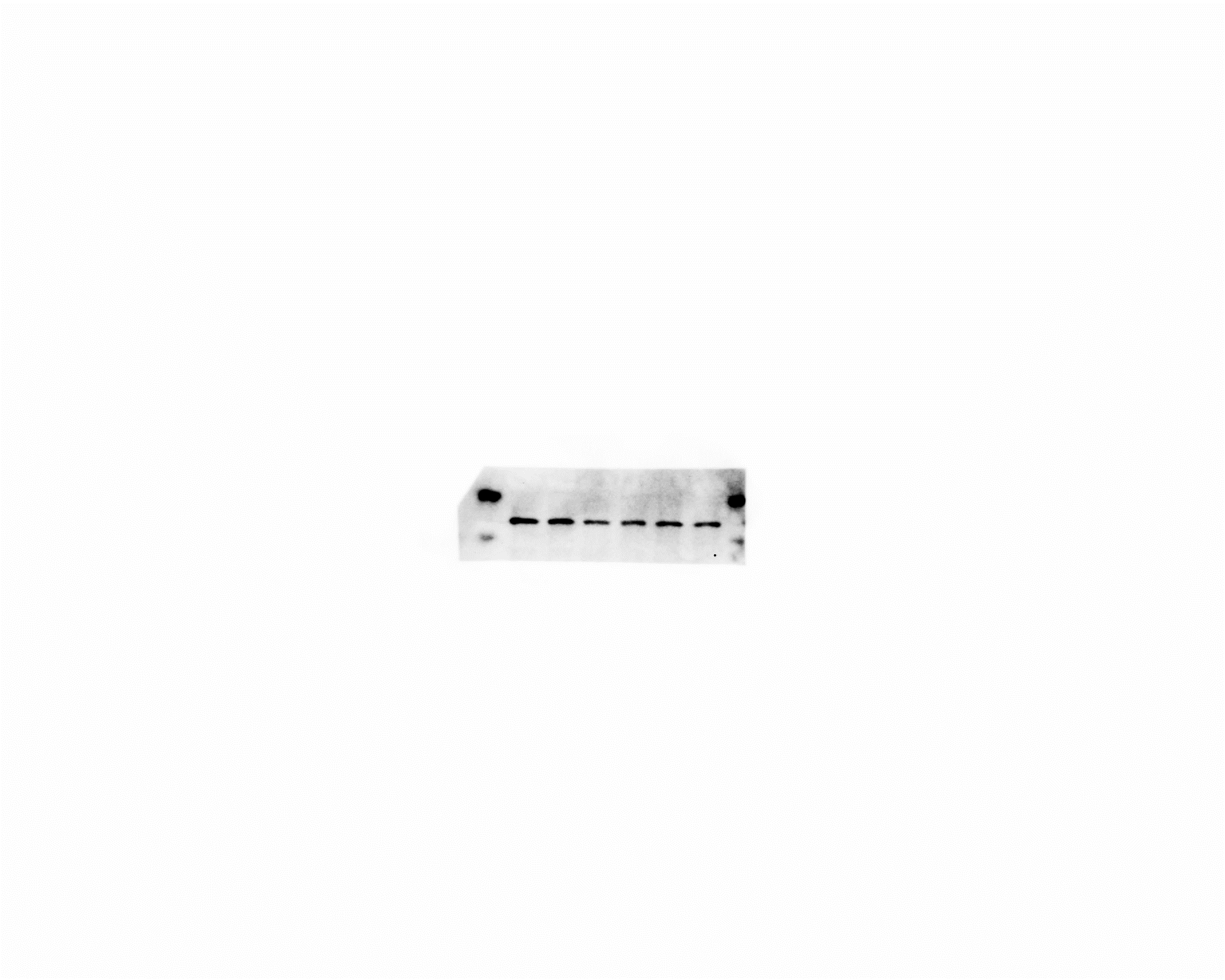

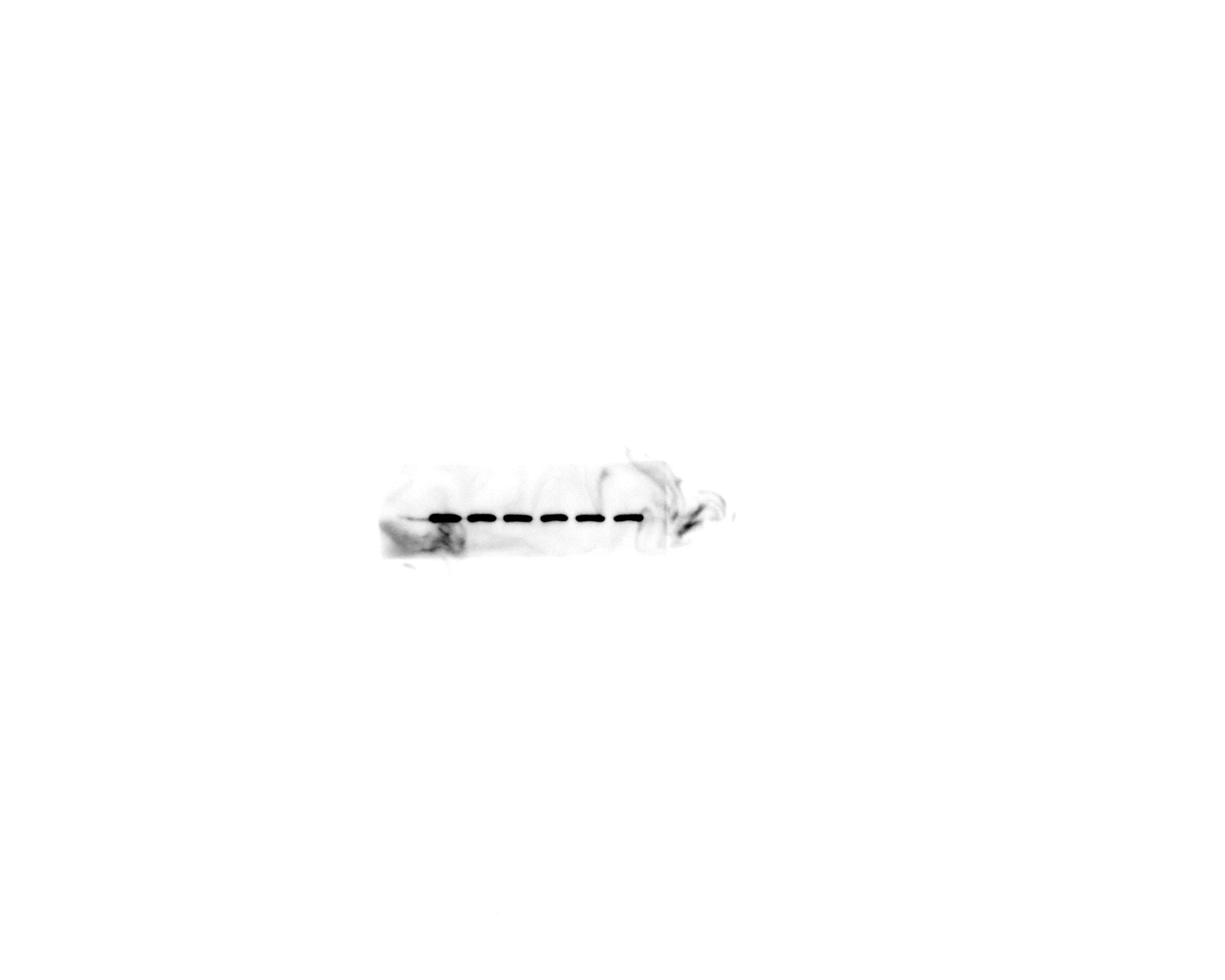


**P53**

**Tubulin**

**TPEN**

**control**

**control**

**TPEN**

**HSYA**

**HSYA**

**+CHX**


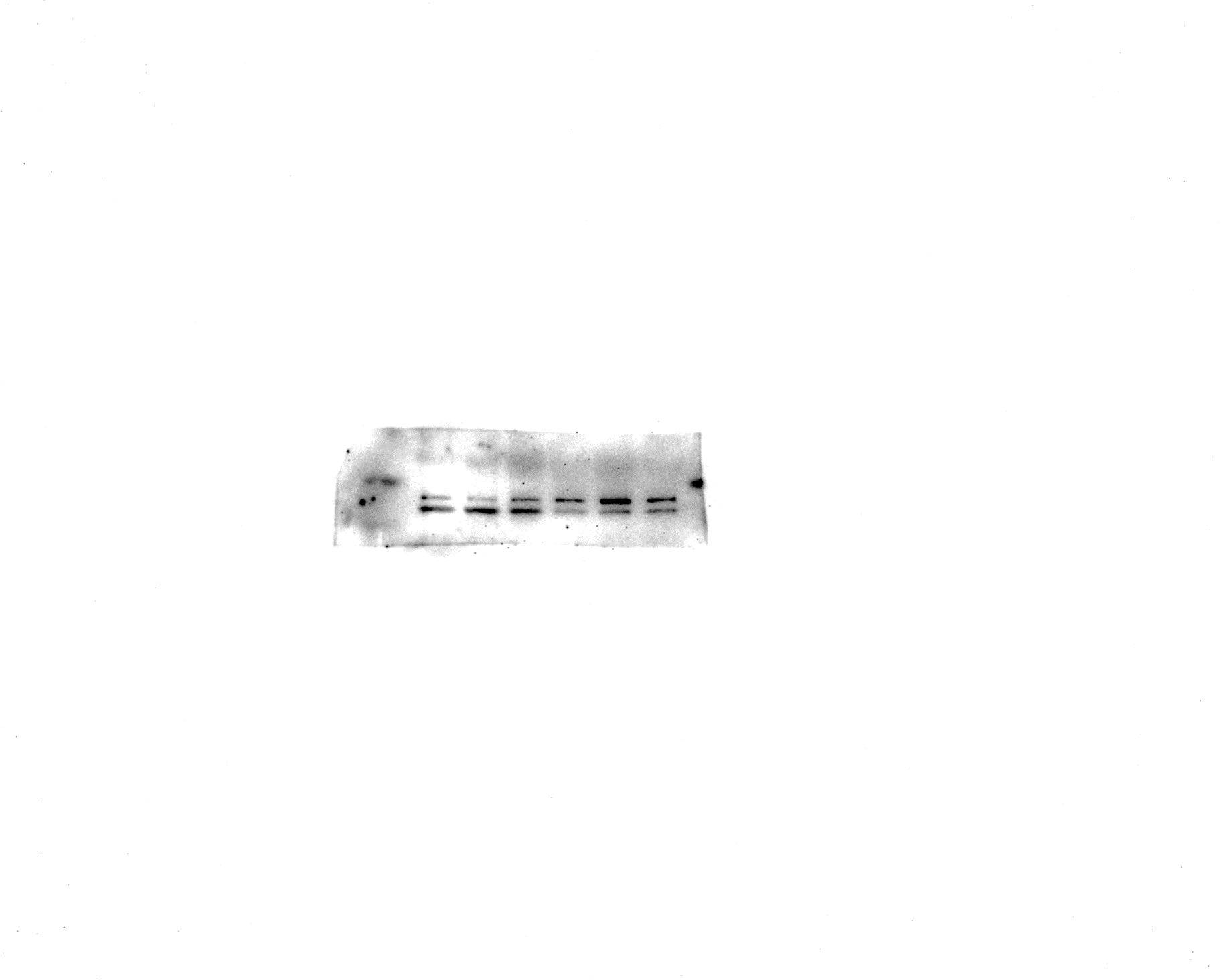

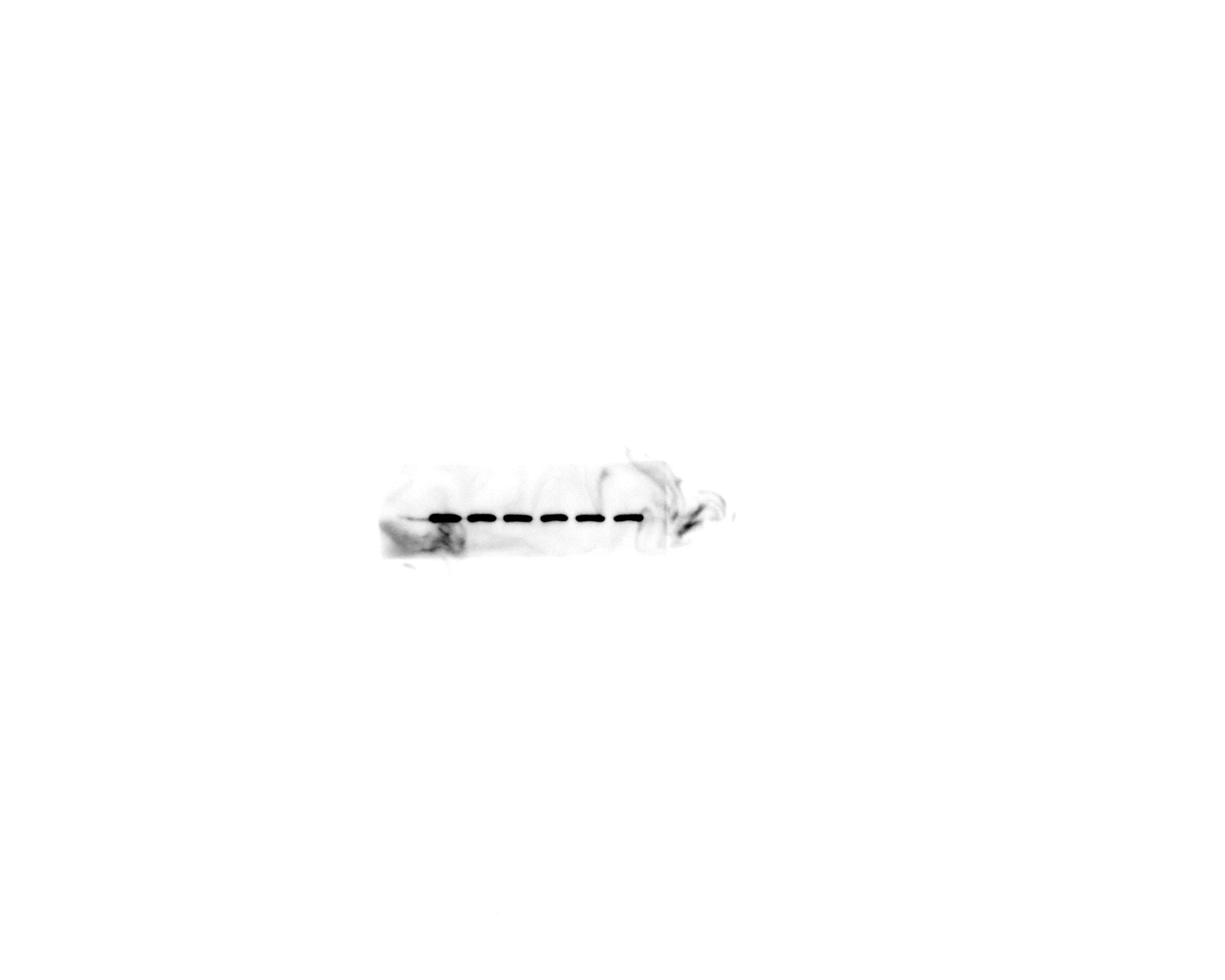


**Figure S3**: Western blot images showing P53 expression. BNCs were cultured in the absence or presence of TPEN(7.5µM), with or without CHX (25 μg/ml) treatment for 3 hours.

**
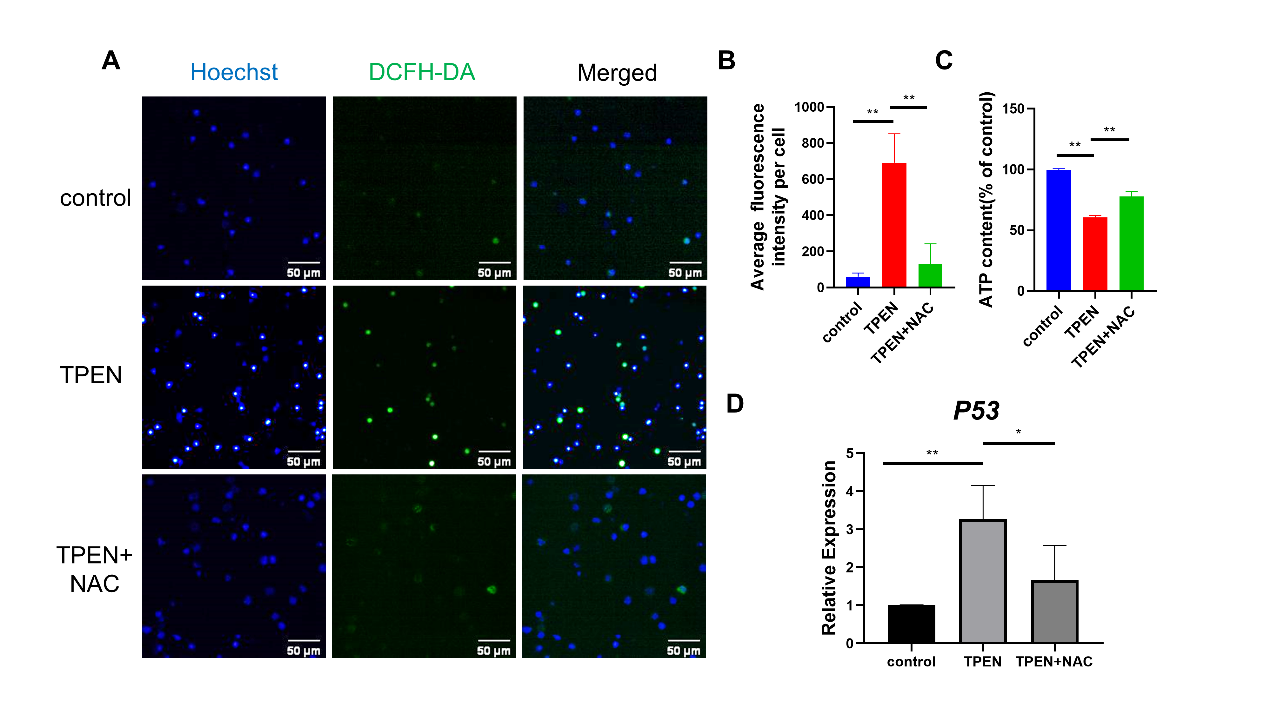
**

**Figure S4:** The rescue effects of ROS inhibitor NAC on TPEN-treated mice bone marrow nucleated cells (BNCs). (A-B) Representative images and quantitative statistics of ROS staining (with DCFH-DA probe) in BNCs. (C) ATP content of control and TPEN-treated BNCs with or without NAC treatment. (D) The level of P53 transcription of control and TPEN-treated BNCs with or without NAC treatment. TPEN (7.5µM), NAC (5mM). Scale bar: 50 µm. *, *P* < 0.05; **, *P* < 0.01.

**Figure S5**: Potential effect of HSYA on the antitumor effect of 5-FU on hepatocellular carcinoma cells HepG2, breast cancer cells MDA-MB-436, colon cancer cells Mc38 and cervical cancer cells Hela, respectively.

**Supplementary Tables S1-S3**

**Table S1: Enriched natural compound library.**

| Compound (CAS) | Herbal Origins | Reported hematopoietic-related functions |
| --- | --- | --- |
| Protopanaxdiol (30636-90-9), Pseudoginsenoside F11 (69884-00-0), Ginsenoside F1 (53963-43-2), Ginsenoside Rd (52705-93-8), Ginsenoside Rh2 (78214-33-2), Ginsenoside Rg2 (52286-74-5), Ginsenoside Re (52286-59-6), Compound K (39262-14-1), Ginsenoside Rg3 (14197-60-5), Ginsenoside Rb3 (68406-26-8), Ginsenoside Rc (11021-14-0), Isoginsenoside Rh3 (166040-90-0), Ginsenoside Rh2 (78214-33-2), Protopanaxdio (30636-90-9) | *Panax ginseng C. A. Meyer* | treatment of aplastic anemia, promotes hematopoiesis, modulates the migration of BMSCs, regulator of hematopoietic stem/progenitor cells, prevention of myelosuppression, prevents BMSCs senescence, improves erythropoiesis in DBA, anti-anemia, regulate hematopoietic homeostasis, enhances hematopoietic function, anti-leukemia, alter macrophage polarization, alleviate cyclophosphamide-induced immunosuppression |
| Isofraxidin (486-21-5) | *Sarcandra glabra* | anti-leukemia, prevention of myelosuppression, promote megakaryocyte proliferation |
| Leonurine hydrochloride (24697-74-3), Syringic acid (530-57-4), Stachydrine hydrochlori (4136-37-2) | *Leonurus japonicus Houtt.* | prevention of hematopoietic damage caused by radiation |
| Crocin II (55750-84-0), Crocin I (42553-65-1), Crocin (42553-65-1) | *Crocus sativus L* | anti-leukemia |
| Schisandrol A (7432-28-2), Schisandrol B (58546-54-6), Schizandrin A (61281-38-7), Schizandrin B (61281-37-6), Schisantherin A (58546-56-8), Schisandrin C (61301-33-5) | *Schisandra chinensis (Turcz.) Baill.* | alleviate cyclophosphamide-induced immunosuppression, promote hematopoietic cell proliferation |
| 1,2,3,4,6-O-pentagalloylglucose (14937-32-7) | *Rhus chinensis Mill.* | protect against radiation-induced damage to the hematopoietic system |
| 4'-O-Glucosylvitexin (38950-94-6) | *Crataegus pinnatifida Bge.* | protect against radiation-induced damage to the hematopoietic system |
| Kaempferol (520-18-3) | *Kaempferia galanga Linn.* | anti-leukemia |
| 20(S)-NotoginsenosideR2 (80418-25-3), Notoginsenoside R1 (80418-24-2) | *Panax notoginseng (Burkill) F. H. Chen ex C. H.* | treatment of aplastic anemia, prevention of myelosuppression, anti-anemia, promote BMSCs mobilization, anti-radiation damage, regulator of hematopoietic stem/progenitor cells |
| Paeonol (552-41-0) | *Paeonia suffruticosaAndr* | prevention of myelosuppression |
| Rosmarinic acid (20283-92-5) | *Rosmarinus officinalis* | anti-leukemia, anti-radiation damage |
| Mangiferin (4773-96-0) | *Mangifera indica L.* | promote BMSCs proliferation |
| (25RS)-Ruscogenin (874485-32-2), Ophiopogonin D' (65604-80-0) | *Ophiopogon japonicus (Linn. f.) Ker-Gawl.* | prevention of myelosuppression |
| Phillyrin (487-41-2) | *Forsythia suspensa* | alleviate cyclophosphamide-induced myelosuppression |
| Amentoflavone (1617-53-4) | *Selaginella tamariscina(Beauv.)Spring* | protect against radiation-induced damage to the hematopoietic system |
| p-Coumaric acid (501-98-4) | *Fagopyrum dibotrys (D. Don) Hara* | alleviate cyclophosphamide-induced myelosuppression |
| Curcumin (458-37-7), 10-Gingerol (23513-15-7), 8-Gingerol (23513-08-8) | *Zingiber officinale Roscoe* | protect against radiation-induced damage to the hematopoietic system |
| Asiaticoside (16830-15-2) | *Centella asiatica (L.) Urban* | suppression of myeloma |
| Wogonoside (51059-44-0), Baicalin (21967-41-9) | *Scutellaria baicalensis Georgi* | treatment of aplastic anemia, anti-leukemia, suppression of myeloma, treatment of myelodysplastic syndrome, anti-radiation damage |
| Astragaloside III (84687-42-3), Astragaloside II (84676-89-1), Isoastragaloside IV (136033-55-1), Ononin (486-62-4), Astragaloside IV (84687-43-4), Formononetin (485-72-3), 9,10－Dimethoxy-pterocarpane－3－O-β-D-glucoside, Methylnissolin (73340-41-7), 3-Hydroxy-9,10-Dimethoxypterocarpan (73340-41-7) | *Astragalus membranaceus (Fisch.) Bunge* | treatment of myelodysplastic syndrome, prevention of myelosuppression, anti-radiation damage, treatment of aplastic anemia, suppression of myeloma, promote BMSCs mobilization, promote aging hematopoietic stem cell proliferation, immunomodulatory activity, anti-leukemia, anti-anemia |
| Resveratrol (501-36-0) | *Reynoutria japonica Houtt.* | suppression of myeloma, promote the migration and differentiation of BMSCs |
| β-Carotene (7235-40-7) | *Daucus carota var. sativa Hoffm.* | anti-radiation damage, treatment of myelodysplastic syndrome |
| Honokiol (35354-74-6) | *Houpoea officinalis (Rehder & E. H. Wilson) N. H. Xia & C. Y. Wu* | suppression of myeloma |
| Benzoic acid (65-85-0) | *Ziziphus jujuba Mill.* | treatment of anemia, immunomodulatory activity, induce erythropoietin expression |
| Rosarin (84954-93-8), Rosavin (84954-92-7), Salidroside (10338-51-9), Herbacetin (527-95-7) | *Rhodiola rosea L.* | induce erythropoietin expression, prevention of myelosuppression, promote BMSCs mobilization, treatment of myelodysplastic syndrome, anti-leukemia, anti-radiation damage, treatment of aplastic anemia |
| Hydroxysafflor yellow A (78281-02-4) | *Carthamus tinctorius L.* | promote BMSCs proliferation |
| Cyanidin-3-O-glucoside (7084-24-4) | *Oryzasatiua* | anti-radiation damage |
| 2,3,5,4'-tetrahydroxyl diphenylethylene-2-o-glucoside (82373-94-2) | *Fallopia multiflora (Thunb.) Harald.* | promote BMSCs proliferation, anti-anemia, anti-radiation damage |
| Glycyrrhizic acid ammonium salt (1407-03-0), (±)-Liquiritigenin (41680-09-5), Glycyrrhizic acid (1405-86-3), Liquiritin (551-15-5), Glycyrrhetinic acid (1449-05-4), Isoliquiritin apioside (120926-46-7), Isoliquiritigenin (961-29-5) | *Glycyrrhiza uralensis Fisch.* | prevention of myelosuppression, suppression of myeloma, treatment of aplastic anemia, promote BMSCs proliferation, ameliorating anemia, anti-leukemia |
| Chlorogenic acid (327-97-9) | *Eucommia ulmoides Oliver* | promote BMSCs proliferation, anti-leukemia, prevention of myelosuppression |
| Sesamoside (117479-87-5), 8-O-Acetyl shanzhiside methyl ester (57420-46-9), Shanzhiside methylester (64421-28-9) | *Lamiophlomis rotata (Benth. exHook. f.) Kudo* | promote proliferation of granulocyte progenitor cells |
| Rehmannioside A (81720-05-0) | *Rehmannia glutinosa (Gaetn.) Libosch. exFisch. et Mey.* | treatment of aplastic anemia, induce erythropoietin expression, prevention of myelosuppression, promote bone marrow cells proliferation, suppression of myeloma, anti-leukemia, ameliorating anemia |
| Salvianolic acid C (115841-09-3), Salvianolic acid B (115939-25-8), Sodium Danshensu (67920-52-9), Danshensu (76822-21-4), TanshinoneⅡA (568-72-9), Lithospermic acid (28831-65-4), Salvianolic acid D (142998-47-8), Salvianolic acid A (96574-01-5), Dimethyl lithospermate B (875313-64-7) | *Salvia miltiorrhiza Bge.* | suppression of myeloma, anti-radiation damage, promote BMSCs proliferation, promote bone marrow stem cells mobilization, prevention of myelosuppression, anti-leukemia, anti-anemia, treatment of myelodysplastic syndrome, treatment of aplastic anemia, anti-radiation damage |
| Chrysophanol (481-74-3), Emodin (518-82-1) | *Rheum palmatum L.* | anti-radiation damage, promote BMSCs proliferation, suppression of myeloma, anti-leukemia |
| Syringin (118-34-3), Eleutheroside E (39432-56-9), | *Acanthopanax senticosus (Rupr. Maxim.) Harms* | anti-radiation damage, prevention of myelosuppression, promote bone marrow microcirculation, promote BMSCs proliferation, immunoenhancement, |
| Senkyunolide I (94596-28-8), Tetramethylpyrazine (1124-11-4) | *Ligusticum chuanxiong hort* | treatment of aplastic anemia, promote hematopoietic microenvironment, promote hematopoietic reconstruction, anti-radiation damage, promote BMSCs proliferation and differentiation |
| (+)-Catechin (154-23-4), (-)-Epigallocatechin/EGC (970-74-1), Epigallocatechin gallate/EGCG (989-51-5) | *tea-leaf* | regulate hematopoietic and immune function |
| Atractylenolide I (73069-13-3), Atractylenolide II (73069-14-4), Atractylenolide III (73030-71-4) | *Atractylodes macrocephala Koidz.* | treatment of aplastic anemia, prevention of myelosuppression, promote BMSCs proliferation and mobilization, immunopotentiation |
| Scopoletin (92-61-5) | *Hedyotis diffusa Willd.* | anti-anemia, treatment of myelodysplastic syndrome, suppression of myeloma, |
| Ferulic acid (1135-24-6) | *Ferula sinkiangensisK. M. Shen* | anti-radiation damage, promote aging hematopoietic stem cell proliferation, prevention of myelosuppression, immunomodulatory activity, promote the apoptosis of leukemia cells |
| Isorhamnetin-3-O-glucoside (5041-82-7) | *Cat-tail Pollen* | treatment of myeloproliferative disorders |
| Calceolarioside B (105471-98-5) | *Akebia quinata (Houtt.) Decne* | promote osteogenic differentiation of bone marrow mesenchymal stem cells |

**Table S2: List of compounds with positive effect on hematopoiesis.**

| Rank | Compound | CAS | Efficacy Score |
| --- | --- | --- | --- |
| 1 | Glycyrrhizic acid ammonium salt | 1407-03-0 | 1.12 |
| 2 | Salvianolic acid C | 115841-09-3 | 1.102332 |
| 3 | Resveratrol | 501-36-0 | 1.024947 |
| 4 | Hydroxysafflor yellow A | 78281-02-4 | 0.952532 |
| 5 | Ferulic acid | 1135-24-6 | 0.92 |
| 6 | Curcumin | 458-37-7 | 0.91 |
| 7 | Pseudoginsenoside F11 | 69884-00-0 | 0.902479339 |
| 8 | Rosarin | 84954-93-8 | 0.9 |
| 9 | Liquiritigenin | 41680-09-5 | 0.88 |
| 10 | Syringin | 118-34-3 | 0.874007937 |

**Table S3: List of QPCR primers used in this study.**

Zebrafish

| Gene | Forward (5’-3’) | Reverse (3’-5’) |
| --- | --- | --- |
| *ef-1α*  (internal reference) | AGAAGGCTGCCAAGACCAAG | AGAGGTTGGGAAGAACACGC |
| *p53* | GCAGCGATGAGGAGATCTTT | GGGCTCAGATGATTCACGAT |
| *p21* | TCACAGATTTCTACCAAGCCAAGA | CGAATGCAGCTCCAGACAGA |
| *bax* | GGCTATTTCAACCAGGGTTCC | TGCGAATCACCAATGCTGT |
| *mdm2* | TAACCGAGGCAGACTACTGGAAG | TTTCCCAGTTGGAGTGTGTTTCT |
| *caspase3* | GAGACCGCTGCCCATCACTAG | ATCCTTTCACGACCATCT |

Mouse

| Gene | Forward (5’-3’) | Reverse (3’-5’) |
| --- | --- | --- |
| *actin*  (internal reference) | GCTGTGCTATGTTGCTCTAG | CGCTCGTTGCCAATACTG |
| *p53* | TGAACCGCCGACCTATCCTT | GCATGGGCATCCTTTAACTCT |
| *bax* | ATGCGTCCACCAAGAAGC | CCAGTTGAAGTTGCCATCAG |
| *caspase3* | TGGGACTGATGAGGAGA | ACTGGATGAACCACGAC |
